# Deciphering the molecular determinants of cholinergic anthelmintic sensitivity in nematodes: When novel functional validation approaches highlight major differences between the model *Caenorhabditis elegans* and parasitic species

**DOI:** 10.1371/journal.ppat.1006996

**Published:** 2018-05-02

**Authors:** Alexandra Blanchard, Fabrice Guégnard, Claude L. Charvet, Anna Crisford, Elise Courtot, Christine Sauvé, Abdallah Harmache, Thomas Duguet, Vincent O’Connor, Philippe Castagnone-Sereno, Barbara Reaves, Adrian J. Wolstenholme, Robin N. Beech, Lindy Holden-Dye, Cedric Neveu

**Affiliations:** 1 ISP, INRA, Université Tours, UMR1282, Nouzilly, France; 2 Biological Sciences, Institute for Life Sciences, University of Southampton, Southampton, United Kingdom; 3 Institute of Parasitology, McGill University, Macdonald Campus, Ste. Anne de Bellevue, Québec, Canada; 4 INRA, Université Côte d'Azur, CNRS, ISA, France; 5 Department of Infectious Disease & Center for Tropical and Emerging Global Disease, University of Georgia, Athens, GA, United States of America; University of Pennsylvania, UNITED STATES

## Abstract

Cholinergic agonists such as levamisole and pyrantel are widely used as anthelmintics to treat parasitic nematode infestations. These drugs elicit spastic paralysis by activating acetylcholine receptors (AChRs) expressed in nematode body wall muscles. In the model nematode *Caenorhabditis elegans*, genetic screens led to the identification of five genes encoding levamisole-sensitive-AChR (L-AChR) subunits: *unc-38*, *unc-63*, *unc-29*, *lev-1* and *lev-8*. These subunits form a functional L-AChR when heterologously expressed in *Xenopus laevis* oocytes. Here we show that the majority of parasitic species that are sensitive to levamisole lack a gene orthologous to *C*. *elegans lev-8*. This raises important questions concerning the properties of the native receptor that constitutes the target for cholinergic anthelmintics. We demonstrate that the closely related ACR-8 subunit from phylogenetically distant animal and plant parasitic nematode species functionally substitutes for LEV-8 in the *C*. *elegans* L-AChR when expressed in *Xenopus* oocytes. The importance of ACR-8 in parasitic nematode sensitivity to cholinergic anthelmintics is reinforced by a ‘model hopping’ approach in which we demonstrate the ability of ACR-8 from the hematophagous parasitic nematode *Haemonchus contortus* to fully restore levamisole sensitivity, and to confer high sensitivity to pyrantel, when expressed in the body wall muscle of *C*. *elegans lev-8* null mutants. The critical role of *acr-8* to *in vivo* drug sensitivity is substantiated by the successful demonstration of RNAi gene silencing for *Hco-acr-8* which reduced the sensitivity of *H*. *contortus* larvae to levamisole. Intriguingly, the pyrantel sensitivity remained unchanged thus providing new evidence for distinct modes of action of these important anthelmintics in parasitic species versus *C*. *elegans*. More broadly, this highlights the limits of *C*. *elegans* as a predictive model to decipher cholinergic agonist targets from parasitic nematode species and provides key molecular insight to inform the discovery of next generation anthelmintic compounds.

## Author summary

Parasitic nematodes have global health and economic impacts. They infect animals, including livestock, humans, and plants including all major food crops. Their control in human and veterinary medicine is reliant on anthelmintic drugs but this is now challenged by resistant worms especially in livestock. Importantly, for anthelmintics such as levamisole and other cholinergic agonists, resistance appears to be less frequent stressing the need to investigate their molecular target in parasitic nematodes. The levamisole receptor was first identified in the free-living model nematode *C*. *elegans* but it is now becoming apparent that this is not a good predictor for many parasitic species. In particular we have found that the LEV-8 subunit which is involved in levamisole sensitivity in *C*. *elegans*, is not present in many levamisole-sensitive parasitic species. Here we used heterologous expression systems and gene silencing to provide the functional *in vivo* demonstration that the ACR-8 subunit, which is not an essential component of the levamisole receptor in *C*. *elegans*, has a critical role in the levamisole sensitivity of parasitic nematodes. This has important significance for understanding the molecular targets of cholinergic anthelmintics and addresses the increasing challenge of drug resistance.

## Introduction

In the absence of efficient alternative strategies, treatment and prophylaxis of parasitic nematode infections rely on anthelmintic compounds. However, resistance against these drugs compromises the sustainable control of highly pathogenic nematode species infecting humans, livestock and companion animals [1–7]. In order to optimize the use of anthelmintic drugs and identify potential molecular mechanisms associated with resistance, there is an urgent need to decipher their pharmacological targets in parasitic nematode species. Among the most widely used anthelmintics, cholinergic agonists such as levamisole and pyrantel are of prime interest as resistance against these drugs is less frequent than for the two other major anthelmintic classes: benzimidazoles and macrocyclic lactones [8–9]. Therefore, the identification and characterization of cholinergic agonist receptors from nematodes have driven important research efforts during the last decades [10–13]. Although nicotinic agonists are not currently used in the treatment or prevention of plant parasitic or filarial worms, recent reports of their *in vitro* efficacy further highlight the importance of elucidating their pharmacological targets [14–18].

Cholinergic agonists bind to acetylcholine receptors (AChRs) which are members of the pentameric Cys-loop ligand-gated ion channels super-family. These receptors play a pivotal role in fast neurotransmission and are widely used at important neuromuscular synapses that control motility and feeding behavior in nematodes. Anthelmintics such as levamisole and pyrantel induce a spastic paralysis of the worm by activating the levamisole-sensitive AChR (L-AChR) expressed in the body-wall muscle cell [19–21]. Taking advantage of the powerful genetic tools available in the model species *C*. *elegans*, screenings for levamisole-resistant mutants led to the identification of the five genes encoding the L-AChR subunits (*unc-38*, *unc-63*, *unc-29*, *lev-1* and *lev-8*) [22–26]. In 2008, Boulin *et al*. demonstrated that combination of these five AChR subunits with a set of three ancillary proteins (UNC-50, UNC-74 and RIC-3) gives rise to a functional L-AChR when co-expressed in the *Xenopus laevis* oocyte heterologous expression system [27]. Levamisole acts as a partial agonist on the recombinant *C*. *elegans* L-AChR, whereas this receptor is insensitive to nicotine [27]. The latter is consistent with evidence showing that nicotinic sensitivity of *C*. *elegans* body wall muscle is conferred by a homomeric receptor comprised of ACR-16 [28]. This first functional expression and pharmacological characterization of the *C*. *elegans* L-AChR provided a strong basis to investigate its parasitic nematode counterparts. Studies of parasitic nematode L-AChRs highlighted some striking differences with the *C*. *elegans* L-AChR [11, 12]. Noticeably, whereas the LEV-8 subunit is a component of the *C*. *elegans* L-AChR, searches in the genomic databanks from *Brugia malayi* (Clade III) and *Trichinella spiralis* (Clade I) failed to identify a *lev-8* ortholog in these parasites [29]. The absence of *lev-8* orthologs was also reported in parasitic nematode species phylogenetically closely related to *C*. *elegans* (Clade V) for which levamisole is successfully used as an anthelmintic (i.e. *Haemonchus contortus*, *Teladorsagia circumcincta*, *Trichostrongylus colubriformis*) and has known spastic potency [30, 31]. Taken together, these observations have prompted the hypothesis that the subunit composition of parasitic nematode L-AChRs would have to differ from the subunit composition from the *C*. *elegans* L-AChR. This has important ramifications for understanding the mode of action of the widely used anthelmintic levamisole and the emergence, monitoring and management of resistance.

In *H*. *contortus*, comparative transcriptomic analysis performed on levamisole-susceptible and levamisole-resistant isolates led to the identification of an AChR subunit (Hco-ACR-8) closely related to the ACR-8 and LEV-8 subunits from *C*. *elegans* [32]. In *C*. *elegans acr-8*, like *lev-8*, is expressed in body wall muscle, although it is not required for a functional levamisole receptor [33]. Using the *Xenopus* oocyte expression system, we previously demonstrated that Hco-ACR-8 is able to associate with the Hco-UNC-63, Hco-UNC-29.1 and Hco-UNC-38 subunits to form a functional AChR (referred to as Hco-L-AChR-1) highly sensitive to levamisole, but relatively insensitive to nicotine and pyrantel [34]. Strikingly, when Hco-ACR-8 was omitted from the cRNA mix, a functional receptor made from Hco-UNC-63, Hco-UNC-29 and Hco-UNC-38 subunits was readily expressed. This second receptor (referred to as Hco-L-AChR-2) was relatively unresponsive to levamisole but highly sensitive to nicotine and pyrantel. Recently, Duguet *et al*. reconstituted two novel L-AChRs of *H*. *contortus*: Hco-L-AChR-1.3 and Hco-L-AChR-1.4. [35]. Both receptors that differed in their subunit composition by the presence of distinct *H*. *contortus* UNC-29 paralogs, contained Hco-ACR-8 and displayed high sensitivity to acetylcholine and levamisole. Using a similar approach, the involvement of the ACR-8 subunit in levamisole sensitivity was further confirmed in the pig parasitic nematode *Oesophagostomum dentatum* recombinant L-AChR [36]. This supports a critical role of the ACR-8 subunit in both levamisole sensitivity and AChR subtype diversity in parasitic nematodes. However, due to the lack of efficient stable transformation tools in parasitic nematodes, the role of their respective ACR-8 subunits in L-AChR composition, pharmacological properties and potential diversity remained to be further explored both *in vitro* and *in vivo*.

In the present study, we report that *acr-8* orthologs are widely distributed in parasitic nematode genomes, which is in sharp contrast to *lev-8* orthologs that could be only identified in a subset of parasitic species. We expressed the ACR-8 subunits from a range of animal and plant parasitic nematode species in *Xenopus* oocytes and showed that they functionally complement the *C*. *elegans* L-AChR missing out the LEV-8 subunit. We expressed *acr-8* from the parasitic nematode *H*. *contortus* in a *C*. *elegans* levamisole resistant *lev-8* null mutant and report that *H*. *contortus* ACR-8 fully rescued the levamisole sensitivity of the transgenic worms. Finally, we optimized the use of RNAi in *H*. *contortus* and demonstrate that AChR subunits from the parasite are amenable targets for gene silencing. Taking advantage of this approach, we provide the first functional demonstration of the ACR-8 subunit involvement in the levamisole sensitivity *in vivo*. Intriguingly, *acr-8* gene silencing in *H*. *contortus* reduced the levamisole but not the pyrantel sensitivity providing new evidence for a discrete difference in their mode of action. Overall, our findings strongly support the hypothesis that in contrast with the model species *C*. *elegans*, the ACR-8 subunit from parasitic nematodes plays a critical role in anthelmintic sensitivity. This has major importance for understanding the molecular targets of the widely used cholinergic anthelmintics and will facilitate drug discovery in this arena.

## Results

### Heterogeneous distribution of *acr-8* and *lev-8* genes in the phylum Nematoda

Using the *C*. *elegans* ACR-8 amino-acid sequence as a query, tBLASTn searches were performed against 68 nematode genomic databases available in WormBase-ParaSite (version 8; http://parasite.wormbase.org/) (S1 Table). This revealed that *acr-8* homologs are present in all the available genomes from the nematode species representing the Clades III, IV and V (20, 17 and 24 species, respectively). Strikingly, when a similar search was performed using the closely related *C*. *elegans* LEV-8 amino-acid sequence as a query, *lev-8* homologs could only be identified in a subset of 21 species out of 68, including 17 Clade V species and the five Ascaridae species from Clade III. Noticeably, no *lev-8* homolog could be found in the 17 genomes of Clade IV species studied. Moreover, no *acr-8*/*lev-8* homolog could be clearly assigned in any Clade I nematode species, although some “*acr-8* related” sequences were identified.

**Fig 1:**
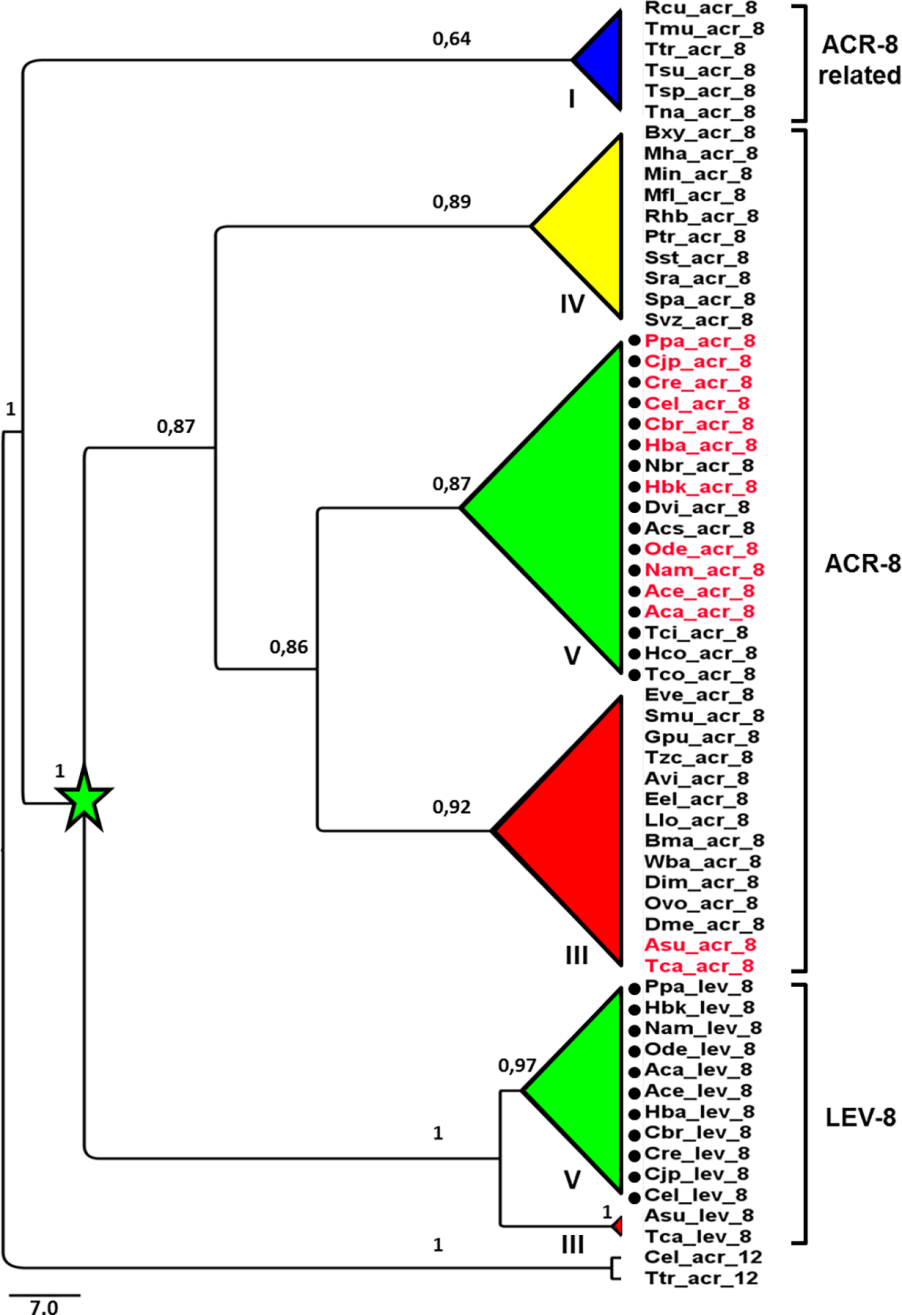
Maximum likelihood phylogeny (PhyML) of *acr-8 and lev-8* codon sequences from Clade I, III, IV and V nematode species. Tree was rooted with the ACR-12 subunit sequences from *Caenorhabditis elegans* and *Trichuris trichiura*. Branch labels correspond to SH values. Scale bar represents the number of substitution per site. The node corresponding to the putative duplication event is indicated by a green star. Nematode species for which a putative *lev-1* homolog could be identified are indicated by a black dot. Nematode species from Clade I, Clade III, Clade IV and Clade V as defined by Blaxter *et al*. [68] are highlighted in dark blue, red, yellow or green respectively. Parasitic nematode species highlighted in red have a *lev-8* homolog. Standard nomenclature indicating the species is provided in Methods section.

Fig 1 shows the orthologous relationships of *C*. *elegans acr-8*/*lev-8* with their counterparts from other nematode species representing Clade I, III, IV and V. This analysis also highlighted that in addition to *acr-8* orthologs, some parasitic nematode species from Clades III (i.e. *Toxocara canis*) and V (i.e. *O*. *dentatum*) possess a *lev-8* ortholog. Importantly, the Clade I nematode *acr*-8 related sequences clustered apart from the *acr-8* and *lev-8* sequences from species belonging to Clades III, IV and V. This suggests that a gene duplication leading to the *acr-8* and *lev-8* genes occurred early in nematode evolution, soon after divergence from the Clade I nematodes.

In addition to the available complete coding *acr-8* sequences from the Clade V species *C*. *elegans*, *H*. *contortus* and *O*. *dentatum* (JF416644, EU006785 and JX429921, respectively), in the present study full-length *acr-8* cDNAs were obtained from other distantly related parasitic nematode species such as the vertebrate parasites *Ascaris suum* (Clade III, pig worm) and *Dirofilaria immitis* (Clade III, dog heart worm) and the plant parasite *Meloidogyne incognita* (Clade IV, root-knot nematode). Based on their respective orthologous relationship with Cel-ACR-8 (Fig 1 and S1 Fig), these novel sequences were named Asu-ACR-8, Dim-ACR-8 and Min-ACR-8 following the recommended nomenclature proposed by Beech *et al*. 2010 [37] and submitted to GenBank under accession numbers: KY654347, KY654349, and KY654350, respectively. All sequences shared typical features of an AChR subunit including a predicted signal peptide, a “Cys-loop”, four transmembrane domains and a YxxCC motif defining them as alpha subunits (S2 Fig). Using the Cel-ACR-8 sequence as reference, mature protein sequences from parasitic species (excluding the signal peptide) shared identities ranging from 58% with Min-ACR-8 to 69% with Hco-ACR-8 or Ode-ACR-8). As expected, sequence identities were lower when compared to Cel-LEV-8 (48–51%) in agreement with Cel-ACR-8 sharing only 45% identity with Cel-LEV-8. Interestingly, as previously reported for the Hco-ACR-8 sequence [34], Asu-ACR-8, Dim-ACR-8 and Min-ACR-8 share common amino acids with Cel-LEV-8 that are not conserved in Cel-ACR-8 (S2 Fig). Such features could therefore suggest a role of these subunits as potential substitute of LEV-8 in the L-AChR.

### Parasitic nematode ACR-8 subunits functionally replace the *C*. *elegans* LEV-8 subunit in the *C*. *elegans* L-AChR expressed in *Xenopus laevis* oocytes

**Fig 2:**
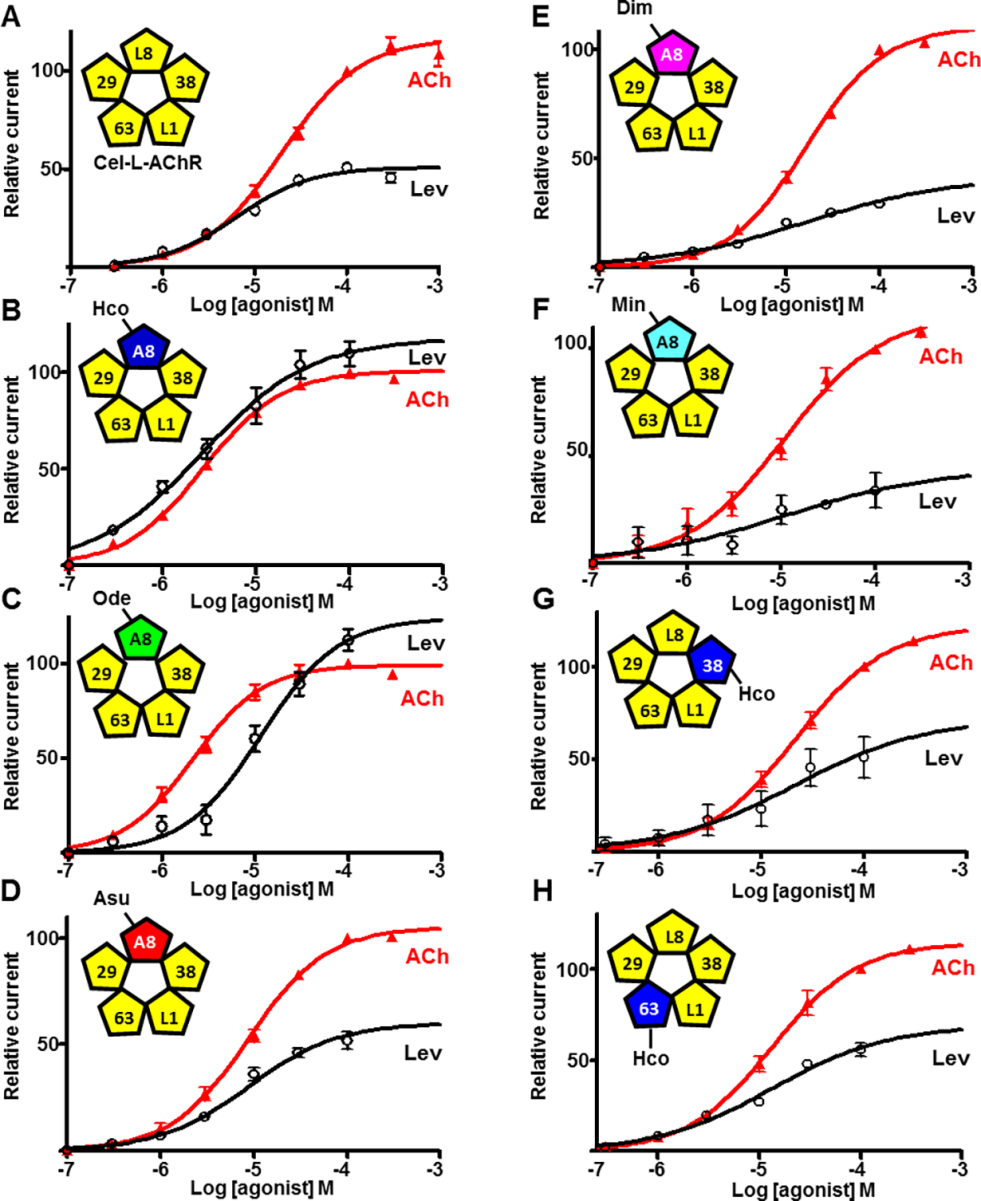
Acetylcholine and levamisole dose response curves for composite L-AChRs containing distinct parasite AChR subunits expressed in *Xenopus* oocytes. **A.** Representation of the putative arrangement of the *C*. *elegans* L-AChR subunits expressed in *Xenopus* oocytes and dose-response relationships for ACh (red triangle, n = 8) and Lev (white circle, n = 8). **B-F.** Representation of the putative subunit arrangement of the composite *C*. *elegans* L-AChRs including Cel-UNC-29, Cel-UNC-38, Cel-UNC-63, Cel-LEV-1 and the ACR-8 subunit from a parasitic nematode species: *H*. *contortus* ACR-8 (**B**), *O*. *dentatum* ACR-8 (**C**), *A*. *suum* ACR-8 (**D**), *D*. *immitis* ACR-8 (**E**) and *M*. *incognita* ACR-8 (**F**). Dose-response relationships for ACh and Lev are indicated by red triangles (n = 5–11) and white circles (n = 4–9), respectively. **G-H.** Representation of the putative subunit arrangement of the composite *C*. *elegans* L-AChRs with Hco-UNC-38 replacing Cel-UNC-38 (**G**) or Hco-UNC-63 replacing Cel-UNC-63 (**H**). Dose-response relationships for ACh and Lev are indicated by red triangles (n = 7–10) and white circles (n = 7–9), respectively. All responses are normalized to 100μM ACh. Results are shown as the mean ± se. EC_50_ and I_max_ values for Ach and Lev are summarized in Table 1.

The *C*. *elegans* L-AChR can be robustly expressed in *Xenopus* oocytes by co-expressing cRNAs from the five L-AChR subunits (UNC-38, UNC-63, UNC-29, LEV-1 and LEV-8) in combination with cRNA encoding three ancillary proteins (UNC-74, UNC-50 and RIC-3) [27]. Noticeably, the absence of any one of these AChR subunits or ancillary proteins from the injected cRNA mix leads to the loss of expression of functional receptor highlighting their major individual requirement. Taking advantage of this, we complemented the Cel-L-AChR by replacing the Cel-LEV-8 by ACR-8 subunits from distinct parasitic nematode species. As a preliminary set of experiments, cRNAs encoding ACR-8 from parasitic nematodes were micro-injected with the three ancillary proteins in order to test their ability to form a homomeric channel. In accordance with previous studies, these experiments failed to produce responses to acetylcholine (ACh) application [34, 36]. Therefore, cRNAs encoding Cel-UNC-38, Cel-UNC-63, Cel-UNC-29, Cel-LEV-1, Cel-UNC-74, Cel-UNC-50 and Cel-RIC-3 in combination with either Hco-ACR-8 / Ode-ACR-8 / Asu-ACR-8 / Dim-ACR-8 / Min-ACR-8 were co-injected into *Xenopus* oocytes. Three days after injection, each of these combinations produced robust responses to 100μM ACh in oocytes (S3 Fig). Similarly, application of 100μM levamisole (Lev) elicited a response for all complemented Cel-L-AChRs. Dose response curves were generated for both ACh and Lev in complemented L-AChRs containing ACR-8 subunits from parasitic species (Fig 2 and Table 1). In comparison to the *C*. *elegans* L-AChR, we observed a drastic reduction of the ACh EC_50_ values in the composite L-AChRs complemented with Hco-ACR-8, Ode-ACR-8, Asu-ACR-8 and Dim-ACR-8. In contrast, when other Cel-L-AChR subunits such as UNC-63 or UNC-38 were replaced by their counterpart from *H*. *contortus* (i.e. Hco-UNC-63 and Hco-UNC-38), we did not observe any modulation of the ACh EC_50_ values (Fig 2 and Table 1). Therefore, these results could support a role of ACR-8 from parasitic species in the ACh sensitivity of composite receptors. Strikingly, for the complemented L-AChR expressing either Hco- or Ode-ACR-8 subunit, Lev acts as a full agonist achieving the same maximum response as ACh induced currents whereas Lev is only a partial agonist of the Cel-L-AChR.

**Table 1:** Summary of the EC_50_ and I_max_ values for acetylcholine, levamisole and pyrantel determined on the *C*. *elegans* L-AChR subtypes and composite receptors expressed in *Xenopus* oocytes.

L-AChR subunit combination		ACh EC_50_ (μM)		Lev EC_50_ (μM)		Lev I_max_	
**29/38/63/L1**	**+ Cel-LEV-8**	**19.6 ±1.1**	*b NS*	**6.4 ±1.2**	*b* ****	**49.1±10.2**	
	**+ Cel-ACR-8**	**20.0±1.1**	*a NS*	**24.6±1.3**	*a* ****	**26.8±1.8**	*a* ***
	**+ Hco-ACR-8**	**2.7 ±1.1**	*a* **** b ****	**2.7 ±1.4**	*a* * b ****	**109.5 ±20.7**	*a* ****
	**+ Ode-ACR-8**	**2.2 ±1.1**	*a* **** b ****	**11.7 ±1.3**	*a* * b ****	**112.4 ±19.0**	*a* ****
	**+ Asu-ACR-8**	**9.0 ±1.1**	*a* **** b ****	**5.8 ±1.2**	*a NS* *b* ****	**51.6 ±7.0**	*a NS*
	**+ Dim-ACR-8**	**10.7 ±1.3**	*a* **** b ****	**5.9 ±1.3**	*a NS* *b* ****	**29.0 ±1.2**	*a* **
	**+ Min-ACR-8**	**16.2 ±1.1**	*a NS* *b NS*	**10.1 ±1.1**	*a* * b ****	**33.9 ±13.9**	*a* *
**29/63/L1/L8**	**+ Hco-UNC-38**	**21.8 ±1.0**	*a NS* *b NS*	**13.5 ±1.5**	*a* ** b ****	**51.1 ±4.5**	*a NS*
**29/38/L1/L8**	**+ Hco-UNC-63**	**17.6 ±1.2**	*a NS* *b NS*	**12.1 ±1.2**	*a* ** b ****	**55.9 ±9.4**	*a NS*
**29/38/63**	**+ Cel-ACR-8**	**18.9±1.1**	*a NS* *b NS*	**51.7±1.4**	*a* **** b ****	**32.3±1.8**	*a* **

The maximum current response was normalized to 100μM ACh. Results are shown as mean ± se.

****p<0.0001

***p<0.001

**p<0.01 and

*p<0.05, one way ANOVA with Bonferroni post-hoc test with 29/38/63/L1+Cel-LEV-8 (a) or 29/38/63/L1+Cel-ACR-8 (b) as reference.

These data suggest that ACR-8 subunits from parasitic nematodes harbor important structural determinants of the Lev response.

### The *C*. *elegans* ACR-8 subunit is a component of novel putative L-AChR subtypes

**Fig 3:**
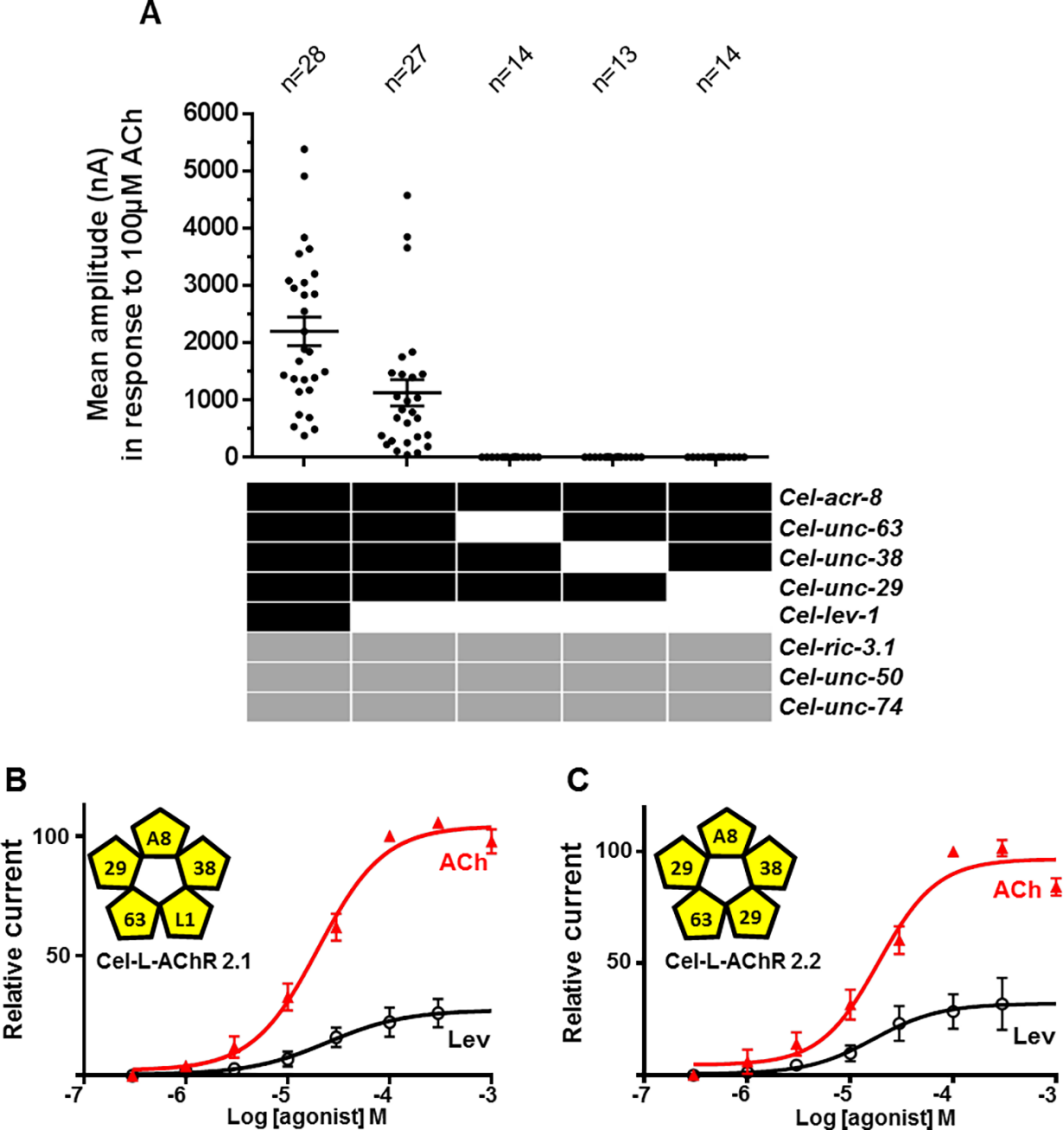
Minimal subunit combination and acetylcholine/levamisole dose response curves for *C*. *elegans* L-AChR subtypes containing the ACR-8 subunit expressed in *Xenopus* oocytes. **A.** The expression of functional AChRs required the co-injection of cRNAs corresponding to ACR-8/UNC-63/UNC-38/UNC-29/LEV-1 or ACR-8/UNC-63/UNC-38/UNC-29 AChR subunits in combination with the three ancillary factors RIC 3; UNC-50 and UNC-74. Scatter plot (mean ± sem) of currents elicited by 100μM ACh. Number of oocytes is reported on the graph. **B-C.** Representation of the putative subunit arrangement of the *C*. *elegans* L-AChR-2.1, (B) and *C*. *elegans* L-AChR-2.2, (C). Dose-response relationships for ACh and Lev are indicated by red triangles (n = 14) and white circles (n = 14) respectively. All responses are normalized to 100μM ACh. Results are shown as the mean ± se. EC_50_ and I_max_ values for Ach and Lev are summarized in Table 1.

Whereas the LEV-8 subunit has been reported as a component of the *C*. *elegans* L-AChR [26, 27, 33], the role of the *C*. *elegans* ACR-8 subunit remained unclear. Here, we investigated the putative functional redundancy of the *C*. *elegans* LEV-8 and ACR-8 subunits using the *Xenopus* oocyte as an expression system. The substitution of LEV-8 by ACR-8 in the recombinant *C*. *elegans* L-AChR led to the robust expression of levamisole-sensitive AChRs (Fig 3 and S3 Fig). This first putative novel *C*. *elegans* L-AChR subtype was named Cel-L-AChR-2.1 (Cel-ACR-8, Cel-UNC-63, Cel-UNC-38, Cel-UNC-29, Cel-LEV-1). Strikingly, the omission of *lev-1* cRNA in the injected mix also led to the robust expression of functional L-AChRs three days after micro-injection (Fig 3 and S3 Fig), mirroring the subunit requirement previously reported for the *H*. *contortus* L-AChR-1 [34] and Ode (38-29-63-8) [36]. This second putative *C*. *elegans* L-AChR subtype was named Cel-L-AChR-2.2 (Cel-ACR-8, Cel-UNC-63, Cel-UNC-38, Cel-UNC-29). In contrast, the omission of UNC-63, UNC-38 or UNC-29 resulted in the absence of functional receptor (Fig 3). Interestingly, whereas Cel-L-AChR, Cel-L-AChR-2.1 and Cel-L-AChR-2.2 share similar ACh EC_50_ values, the receptors including the ACR-8 subunit had higher Lev EC_50_ values in comparison with the receptor including the LEV-8 subunit (Table 1). Importantly, we also observed that composite receptors containing ACR-8 subunits from a parasitic species harbored decreased Lev EC_50_ values in comparison with Cel-L-AChR-2.1 (Table 1). In addition to a decreased sensitivity to Lev, the Cel-L-AChR-2.1 and Cel-L-AChR-2.2 receptors also harbored lower Lev Imax values in comparison with the prototypical Cel-L-AChR highlighting a reduced potency of Lev on the *C*. *elegans* L-AChRs including the ACR-8 subunit (Table 1). The respective role of Cel-ACR-8 and Cel-LEV-8 in Lev sensitivity was further explored in the recombinant *H*. *contortus* L-AChR-1 receptor by substituting Hco-ACR-8 by the *C*. *elegans* subunits (S4 Fig). In both cases, the robust expression of functional composite receptors was obtained. Interestingly, the composite receptor containing Cel-LEV-8 was more responsive to Lev than its counterpart including the Cel-ACR-8 subunits.

Taken together, these results provide strong evidence that both ACR-8 and LEV-8 can contribute to different subtypes of L-AChR in *C*. *elegans* and further support the role of ACR-8 as a key component of Lev sensitivity modulation in nematodes.

Interestingly, we also found that Pyr was more potent on Cel-L-AChR-2.1 and Cel-L-AChR-2.2 than in the prototypical Cel-L-ACh, suggesting that ACR-8 could contribute to Pyr sensitivity in *C*. *elegans* (S5 Fig). In agreement with this assumption, the composite Hco-L-AChR-1 with Cel-ACR-8 replacing Hco-ACR-8 was more responsive to Pyr than the parental Hco-L-AChR-1 receptor (S5 Fig).

### Expression of *Hco-acr-8* in *C*. *elegans lev-8* null mutant rescues levamisole sensitivity

Recently, we showed that *C*. *elegans* was a tractable model to express AChR receptors from parasitic nematode species [35, 38, 39]. Therefore, to address the functional relationship between ACR-8 and LEV-8, we conducted an *in vivo* characterization of the parasitic nematode ACR-8 using *C*. *elegans* as an expression platform. Because we demonstrated that Hco-ACR-8 was able to functionally replace the *C*. *elegans* LEV-8 subunit in the L-AChR expressed in the *Xenopus* oocyte, we took advantage of the *C*. *elegans* model to investigate the ability of Hco-ACR-8 to restore Lev sensitivity in a *lev-8* mutant background. In the present study, we used the *lev-8* null mutant strain VC1041 (*ok1519*) that is resistant to Lev [26]. As a preliminary experiment, expression of GFP driven by the putative *Hco-acr-8* promoter and mCherry driven by the muscle cell specific *C*. *elegans myo-3* promoter was monitored in *C*. *elegans* N2 (wild type). Both *PHco-acr-8* and *Pmyo-3* showed a similar expression pattern in body wall muscle cells (S6 Fig). Thus, the *myo-3* promoter was used to express the Hco-ACR-8 subunit in the *C*. *elegans lev-8*.

**Fig 4:**
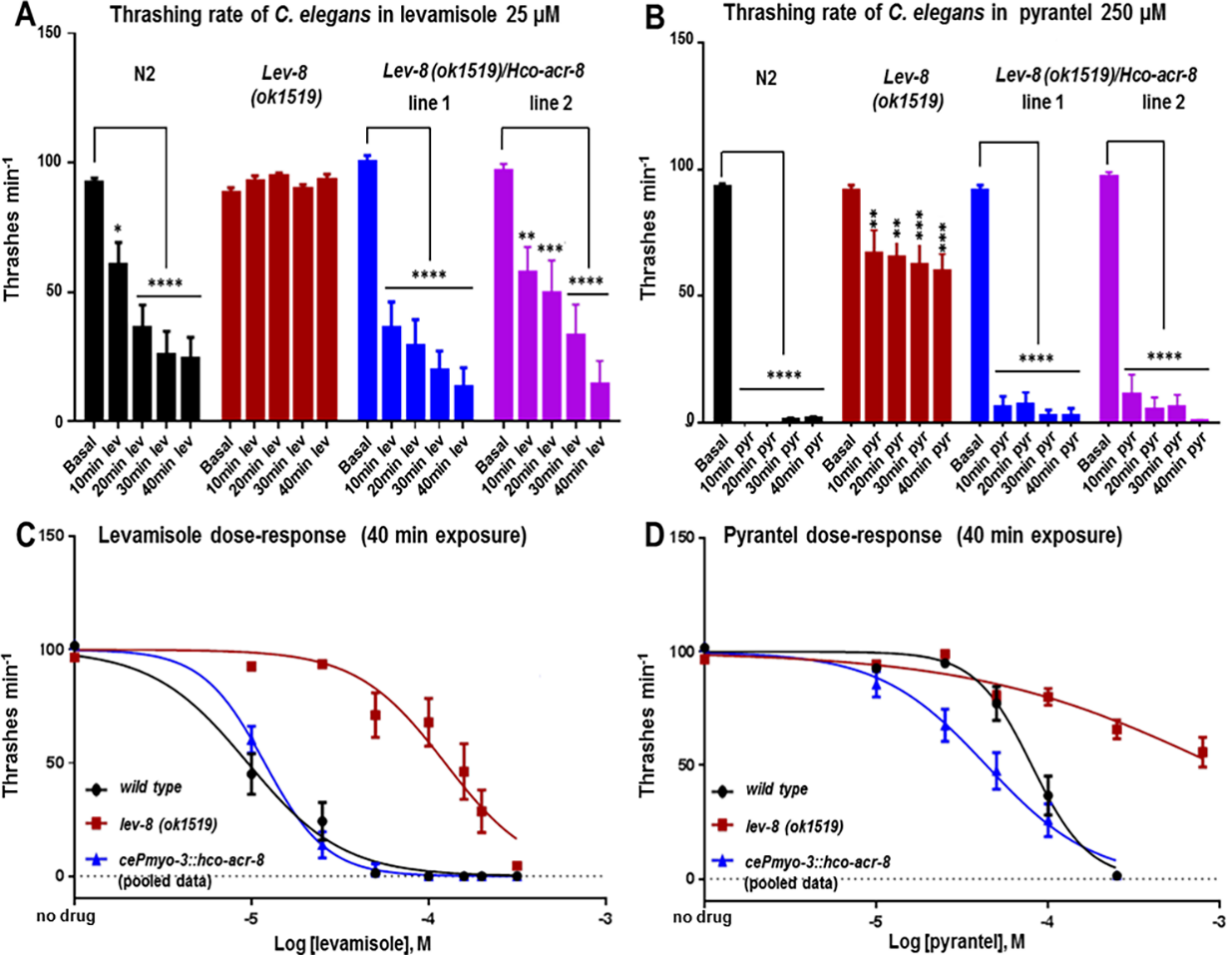
Effects of levamisole and pyrantel on the thrashing rate of *lev-8(ok1519)* expressing *H*. *contortus acr-8*. **A-B.** The thrashing rate was established for wild type N2, *lev-8(ok1519)* and two lines of transgenic *lev-8(ok1519); Pmyo-3*::*hco acr-8 C*. *elegans* after 10, 20, 30 and 40 min of exposure to Lev 25μM (**A**) or Pyr 250μM (**B**), respectively. Basal thrashing rate was established after 10 min acclimatisation in M9 buffer. Data are the mean ± SEM of n = 12, ****p<0.0001, ***p<0.001, **p<0.01 and *p<0.05, one way ANOVA with Bonferroni post-hoc test between basal and after drug treatment thrashing rate for the same strain. **C-D**. Dose response curves for wild type N2, *lev-8(ok1519)* and *lev-8(ok1519); Pmyo-3*::*hco acr-8 C*. *elegans* after 40 min of exposure to 0, 10, 25, 50, 100, 150 and 200 μM Lev (**C**) or Pyr (**D**). Two stable lines for *lev-8(ok1519); Pmyo-3*::*hco acr-8* were tested and the data is pooled. Data are the mean ± SEM of n ≥8. IC_50_ for Lev in N2: 9.5μM (95% confidence limits 7.4 to 12.3μM; n = 8), in *lev-8* null mutants: 124μM (95% confidence limits 94 to 163μM; n = 8) and in *lev-8* null mutants expressing *Hco-acr-8*: 11.9 μM (95% confidence limits 11.4 to 12.3μM; n = 8). IC_50_ for Pyr in N2: 81μM (95% confidence limits 69.2 to 94.9μM; n = 12 to 18) and in *lev-8* null mutants expressing Hco-ACR-8: 44μM. (95% confidence limits 35.4 to 54μM; n = 12). The IC50 for *lev-8* null mutant was not determined.

The motility of the N2 strain and the *lev-8* null mutants (with or without the expression of Hco-ACR-8 in body wall muscle) was very similar, as scored from the thrashing rate in liquid (S6 Fig). Thus, neither the ectopic over-expression of Hco-ACR-8 in body wall muscle, nor the deficiency of *lev-8* impacted the *in vivo* bioassay used in our experiments to report on drug modulation of motility. From this stable background, we were able to investigate the relative Lev sensitivity of N2, mutant and *C*. *elegans* transgenic lines by performing time dependent thrashing inhibition assays with Lev concentrations ranging from 10 to 200μM (Fig 4 and S7 Fig).

As expected, after 40 min exposure to Lev 25μM, N2 worms showed a strong inhibition of thrashing whereas the *lev-8* null mutants were not inhibited. Strikingly, at the same dose and same time point, the *lev-8* null mutant expressing Hco-ACR-8 in the body wall muscle (*lev-8(ok1519); cePmyo-3*::*hco-acr-8)* showed a drastic reduction in thrashing rate (Fig 4). An analysis of the Lev concentration-dependent inhibition of thrashing confirms that the expression of the Hco-ACR-8 subunit functionally complements the *C*. *elegans* L-AChR lacking the LEV-8 subunit and fully restores the Lev sensitivity in the transgenic worms. In view of the results showing that Hco-ACR-8 can assemble with L-AChR subunits in *Xenopus* oocytes, these data suggest that Hco-ACR-8 can act in a similar fashion in *C*. *elegans in vivo* to confer sensitivity of the body wall muscle to Lev on an otherwise Lev resistant strain.

In *C*. *elegans*, functional null mutants for *unc-38*, *unc-63* or *unc-29* genes are resistant to high concentrations of pyrantel (Pyr) suggesting that this anthelmintic could target the L-AChR [40, 41]. However, there was to our knowledge no available data concerning the Pyr resistance status of *lev-8* null mutants. Therefore, we compared the thrashing rate of N2 and *lev-8* null mutants after 10, 20, 30 or 40 min exposure time to Pyr concentrations ranging from 10 to 250μM (Fig 4 and S7 Fig). Whereas N2 worms were completely paralyzed after 10 min incubation with 250μM Pyr, the *lev-8* null mutants only showed a partial reduction of their thrashing rate (Fig 4). This result indicated that the loss of a functional LEV-8 subunit leads to a reduction of Pyr sensitivity in *C*. *elegans*. The ability of *Hco-acr-8* to restore Pyr sensitivity in the *lev-8* null mutant was also investigated. Incubation with increasing concentrations of Pyr led to the paralysis of both N2 and transgenic worms demonstrating that parasitic nematode ACR-8 is able to restore Pyr sensitivity to the *lev-8* null mutant (Fig 4 and S7 Fig). Interestingly, Pyr dose-response assays revealed an increased sensitivity to Pyr in transgenic worms expressing Hco-ACR-8 in comparison to wild-type *C*. *elegans*.

### RNAi silencing of *Hco-acr-8* gene expression in *H*. *contortus* larvae confers levamisole resistance

The above experiments using recombinant approaches in *Xenopus* oocyte or utilizing heterologous subunit expression in *C*. *elegans* highlight a potential role for parasitic nematode ACR-8 in the L-AChRs and Lev sensitivity. In order to further investigate *in vivo* the function of ACR-8, our objective was to optimize the use of RNAi for the silencing of L-AChR subunit genes in *H*. *contortus* and characterize the resulting phenotypes. Gene silencing in *H*. *contortus* L3 larvae has been reported to be challenging because of the unreliable siRNA uptake of the worms [42]. To overcome this limitation, we reasoned that *H*. *contortus* L2 larvae that actively feed from their environment could represent an appropriate alternative to optimize the delivery of double stranded siRNA into the parasite by soaking (S1 Video). The relevance of using *H*. *contortus* L2 stage in our study is supported by two significant observations: firstly, *H*. *contortus* L2 are highly sensitive to Lev and secondly, RT-PCR experiments confirmed the expression of the AChR subunits that constitute the Hco-L-AChR-1 in this developmental stage (S8 Fig).

In order to investigate the potential ingestion of RNA constructs for gene silencing experiments, *H*. *contortus* L2 larvae were incubated in a culture medium containing 1μM of a non-specific siRNA targeting *gfp* (non-specific target) labeled with the fluorochrome Alexa-594 (S8 Fig). After an incubation time of 2h, strong fluorescent signals were observed in the esophagus and the intestinal lumen of all the *H*. *contortus* L2 larvae providing an indication of the robust siRNA uptake by the nematode. Notably, visual scoring showed that *H*. *contortus* larvae retained normal viability even after 96 hours of siRNA treatment indicating an absence of toxicity from the ingested siRNA. Subsequently, *H*. *contortus* L2 larvae were incubated with siRNA targeting *Hco-unc-38*, *Hco-unc-63* or *Hco*-*acr-8*, respectively. After 72h of siRNA incubation, the silencing of the *Hco-unc-63* and *Hco-unc-38* genes resulted in uncoordinated locomotion in at least 50% of the larvae illustrating a key role for L-AChR subunits UNC-63 and UNC-38 in cholinergic neuromuscular transmission (S2–S4 Videos). These observations are consistent with the uncoordinated phenotype in *C*. *elegans* carrying mutation in these subunits [22]. These data indicated that L-AChR subunits are amenable to gene silencing in *H*. *contortus*. Also, in accordance with the phenotype of *C*. *elegans acr-8* (or *lev-8*) null mutants [26, 33], 72h incubation with double strand siRNA targeting *Hco-acr-8* did not impact on the motility of *H*. *contortus* larvae (S5 Video).

**Fig 5:**
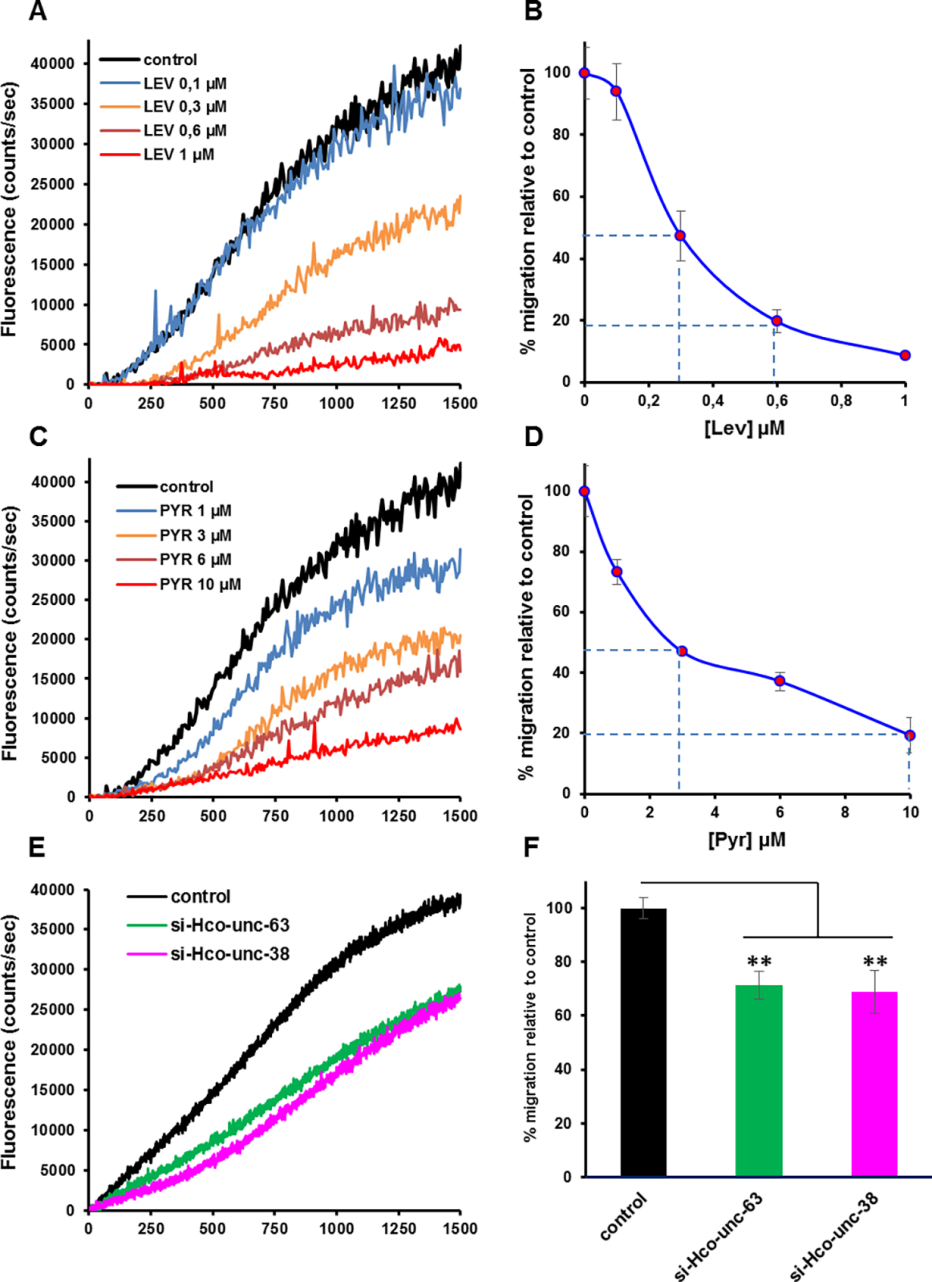
Motility modulation of *H*. *contortus* L2 larvae exposed to cholinergic agonists or siRNA targeting AChR subunits. The automated larval migration assay (ALMA) was used to determine dose-dependent paralysis effect of Lev and Pyr and motility reduction associated with the silencing of *Hco-unc-38* and *Hco-unc-63* respectively. **A and C.** Representative recording traces of the real-time fluorescence counting relative to the L2 migration during 25min exposed to Lev (**A**) or Pyr (**C**). Each trace corresponds to the mean data from 3 runs performed with 7500 L2 larvae. **B and D.** Dose response relationships for Lev (**B)** and Pyr (**D**). Results are shown as the mean ± se. **E.** Effect of siRNA targeting *Hco-unc-38* or *Hco-unc-63* on the motility *H*. *contortus* L2 larvae. Each curve corresponds to the mean data from 3 distinct assays performed with 7500 L2 larvae. The control corresponds to untreated L2 larvae. **F.** Estimation of the motility reduction of L2 larvae exposed to siRNA targeting *Hco-unc-38* or *Hco-unc-63* relative to control. Mean data of the ten last fluorescence measures ± SE. **p<0.01, one way ANOVA with Bonferroni post-hoc test between untreated and siRNA treated worms from the same isolate.

**Fig 6:**
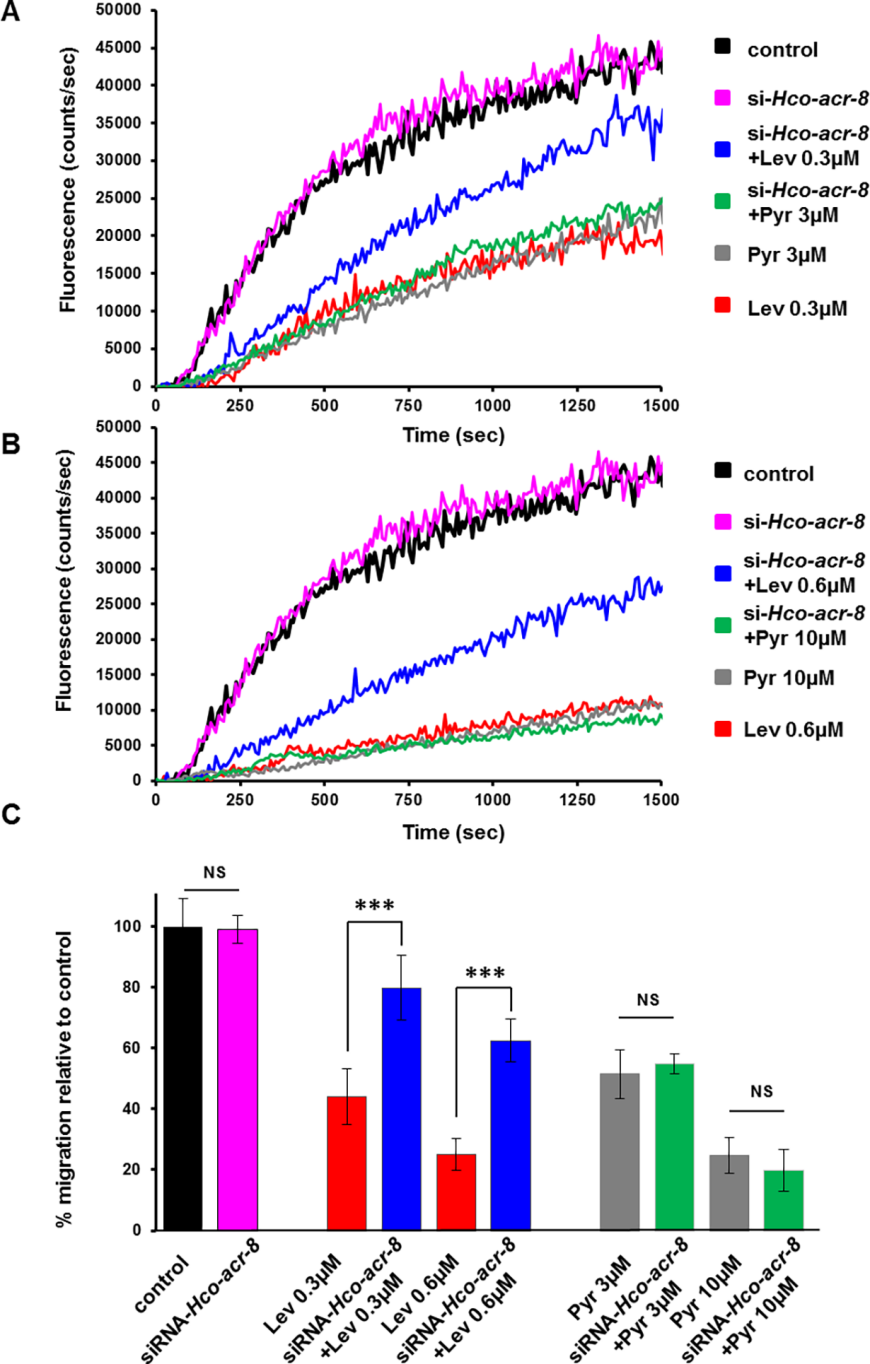
Effects of *Hco-acr-8* silencing on levamisole sensitivity of *H*. *contortus* L2 larvae. The automated larval migration assay (ALMA) was used to determine modulation of Lev or Pyr sensitivity in L2 larvae exposed to siRNA targeting *Hco-acr-8*. **A-B.** Representative recording traces of the real-time fluorescence counting relative to the L2 migration during 25min exposed to Lev 0.3μM or Pyr 3μM (**A**) or Lev 0.6μM or Pyr 10μM (**B**). The control corresponds to untreated L2 larvae. Each trace corresponds to the mean data from 3 runs performed with 7500 L2 larvae. **C.** Estimation of the paralysis reduction of siRNA-treated L2 larvae exposed to Lev or Pyr. Mean data of the ten last fluorescence measures ± SE. ***p<0.001, one way ANOVA with Tukey’s multiple comparisons test.

In order to better define the motility deficiencies resulting from the *H*. *contortus* L-AChR subunit silencing, we developed an automated larval migration assay. Taking advantage of the natural auto-fluorescence of the *H*. *contortus* L2 larvae, we designed a spectrofluorometric-based approach to measure real-time fluorescence correlated with the migration rate of the worms (S8 Fig). The correlation coefficient R^2^ showed a highly significant relationship between the fluorescence measured and the number of larvae that migrated through the sieve (R^2^ = 0.9935) after 25 min. Therefore, we were able to automate the measurement of larval migration, and the effect of anthelmintics on this behaviour, by recording the increase in fluorescence against time in the absence or presence of drug. Paralysis assays performed on *H*. *contortus* L2 stage with Lev or Pyr confirmed the suitability of this approach to parameterize the effects on motility (Fig 5). Using this assay, we confirmed a reduction of the motility of *H*. *contortus* L2 larvae incubated with siRNA targeting *Hco-unc-63* and *Hco-unc-38* AChR subunit transcripts (Fig 5). In contrast, the motility of *H*. *contortus* L2 larvae incubated with siRNA targeting *Hco-acr-8* was similar to control worms (Fig 6). Therefore, as previously reported for *C*. *elegans*, the AChR subunits UNC-63 and UNC-38 are involved in the control of motility in *H*. *contortus*, whereas ACR-8 is not required for wild type locomotion [33]. The potential modulation of Lev sensitivity associated with *Hco-acr-8* gene silencing was investigated in *H*. *contortus* L2 larvae using Lev concentrations corresponding to the minimal efficient doses leading to 50 and 80% of motility reduction respectively (i.e. Lev 0.3μM and Lev 0.6μM) (Fig 6). Strikingly, for both Lev concentrations, *Hco-acr-8* silenced larvae showed a reduction of Lev sensitivity in comparison with the control larvae not subjected to silencing. This provides the first *in vivo* evidence for a key role of ACR-8 in the Lev sensitivity of a parasitic nematode. Intriguingly, whilst *Hco-acr-8* siRNA silenced larvae showed a reduced sensitivity to Lev, they were still subject to the inhibitory effects of Pyr. Indeed, using either Pyr 3μM or Pyr 10μM corresponding to the minimal efficient dose leading respectively to 50% or 80% of motility reduction in *H*. *contortus* L2, the migration of *Hco-acr-8* siRNA silenced larvae was similar to control worms (Fig 6). These data suggest that Lev and Pyr could mediate their inhibitory effects on *H*. *contortus* motility via two distinct pharmacological targets.

## Discussion

### ACR-8 is a key determinant of levamisole sensitivity for parasitic nematodes

Screens for *C*. *elegans* mutants that resist to Lev have been instrumental to identify the 5 AChR subunits constituting the L-AChR (i.e. UNC-38, UNC-63, UNC-29, LEV-1 and LEV-8) [43] and laid a strong basis to investigate their counterpart in parasitic species. However, the absence of *lev-8* homologs in closely related parasitic species for which Lev is widely used as an anthelmintic raised the question of an alternative L-AChR subunit composition in these nematodes. In the present study we demonstrate that ACR-8, the closest homolog of the *C*. *elegans* LEV-8 subunit, can play a pivotal role *in vitro* and *in vivo* in the composition and pharmacological properties of L-AChR from parasitic nematodes.

The analysis of genomic data from 68 nematode species raised the question of the origin of *acr-8* and *lev-8* homologs within the Nematoda. The phylogenetic analyses show that the *acr-8* and *lev-8* genes result from an early gene duplication event that occurred after divergence of the Clade I with the other nematode clades. It is noteworthy that Clade I species such as *Trichinella spiralis*, *Trichuris suis* or *Trichuris muris* are sensitive to Lev [44–46] providing evidence that in contrast with the *C*. *elegans* L-AChR, the LEV-8 subunit is not involved in the functional Lev receptors of these parasitic species.

Since *lev-8* must have been present in the common ancestor of Clade III, Clade IV and Clade V nematodes, the absence of *lev-8* in species from these clades strongly suggests independent events where *lev-8* was lost. Even though we cannot rule out that incomplete genomic data could explain the failure to identify *lev-8* orthologs in some species from Clade III, Clade IV and Clade V, the systematic identification of *lev-8* homolog in species harboring an *acr-8* homolog as well as common amino acid signature shared by the *C*. *elegans* LEV-8 and ACR-8 sequences from parasitic species support the hypothesis that *lev-8* loss was mediated by distinct evolutionary events.

### Composite nematode L-AChR expressed in *Xenopus laevis* oocytes as a tool to decipher parasitic nematode ACR-8 subunit functionality

The substitution of the *C*. *elegans* LEV-8 subunit by ACR-8 from animal or plant parasitic nematode species in the recombinant *C*. *elegans* L-AChR (Cel-L-AChR) led to the robust expression of functional receptors. Interestingly, we showed that ACR-8 from *A*. *suum* and *O*. *dentatum*, species that also possess a *lev-8* homolog, also functionally complement the Cel- L-AChR lacking the LEV-8 subunit. These results highlight the need for future research in order to investigate the putative functional redundancy between ACR-8 and LEV-8 in these two distantly related species. Interestingly, such redundancy is apparent in *C*. *elegans* as we demonstrated that the *C*. *elegans* ACR-8 subunit can functionally substitute the LEV-8 subunit in the recombinant Cel-L-AChR. In accordance with previous molecular and electrophysiological data that support the involvement of Cel-ACR-8 in a putative subset of L-AChR channels in *C*. *elegans*, here we report the first functional evidence for novel L-AChR subtypes from *C*. *elegans* (Cel-L-AChR-2.1 and Cel-L-AChR-2.2) containing the ACR-8 subunit [33, 47]. These results lay the basis to decipher the role of alternative Cel-L-AChR in the synaptic function and their potential interactions with accessory proteins specific to L-AChR such as MOLO-1 [48].

In previous studies performed on recombinant L-AChRs from *H*. *contortus* and *O*. *dentatum* expressed in *Xenopus* oocytes, we reported that ACR-8 is a critical subunit for the Lev response of Hco-L-AChR-1 and Ode (38-29-63-8), respectively [34, 36]. In the present work, when the *C*. *elegans* LEV-8 subunit was substituted by either Hco-ACR-8 or Ode-ACR-8 in the *C*. *elegans* L-AChR, Lev acted as a full agonist on the composite receptors as previously reported for Hco-L-AChR-1 and Ode (38-29-63-8), respectively [34, 36]. In contrast, on the *C*. *elegans* recombinant L-AChR, Lev acts only as a partial agonist. Indeed, Lev I_max_ values of the composite receptors containing Hco-ACR-8 (109.5±20.7) or Ode-ACR-8 (112.4±19.0) are within the same range as for Hco-L-AChR-1 (119.9±16.8) and Ode (38-29-63-8) (119±3.7) respectively [36] whereas Lev I_max_ for the Cel-L-AChR is 49.1±10.2. Interestingly, when either Cel-UNC-63 or Cel-UNC-38 subunit was substituted by its *H*. *contortus* counterpart, the Lev I_max_ value was not modified in comparison with the “native” recombinant Cel-L-AChR. Similarly, the replacement of Cel-UNC-29 by UNC-29 paralogs from *H*. *contortus* in the Cel-L-AChR does not modulate the Lev response of the resulting composite receptors [35]. Therefore, it is tempting to speculate that ACR-8 from both parasitic nematode species could be directly involved in the Lev response of the composite receptor. Because ACR-8 subunits from parasitic species expressed in Cel-L-AChR lacking LEV-8 could potentially recapitulate the Lev sensitivity of their native L-AChR, this experimental platform could provide a basis for an original target-based drug screening tool. In order to test the relevancy of such an approach, functional reconstitution of L-AChRs from other parasitic nematode species is now urgently needed.

Of note, is the particular case of *A*. *suum* for which co-expression in *Xenopus* oocyte of UNC-38 and UNC-29 subunits led to functional Lev-sensitive receptors [49]. In the present study, we demonstrate that ACR-8 from *A*. *suum* functionally complements the Cel-L-AChR lacking LEV-8, resulting in a Lev-sensitive AChR. In *A*. *suum*, *in vivo* electrophysiological studies revealed three distinct AChR subtypes responsive to Lev [50]. In that respect, further investigation of *A*. *suum* L-AChRs that incorporate the ACR-8 subunit would be of particular interest in order to decipher the potential L-AChR subtype diversity for *A*. *suum*.

Taken together, our results provide novel functional evidence for a critical role of the ACR-8 subunit in determining the Lev sensitivity of the parasitic nematode L-AChR and further validate the composite AChR approach to delineate the discrete role of parasitic nematode subunits in receptor function and pharmacology.

### *C*. *elegans lev-8* null mutants as a tool to decipher the role of ACR-8 from parasitic nematode species

In the present study, in order to investigate the specific function of ACR-8 subunit from parasitic nematodes, we decided to use *C*. *elegans lev-8* null mutant genetic background for the following reasons:

First, in *C*. *elegans* both *lev-8* and *acr-8* null mutants show no significant changes in thrashing rate with respect to N2 (wild type) worms [33]. However, whereas *lev-8* null mutants are partially resistant to Lev, *acr-8* mutants are not resistant to this drug [26, 33]. Second, split tandem affinity purification performed in *C*. *elegans* with UNC-29/LEV-1 as baits resulted in the identification of ACR-8 as a co-assembled subunit [47].

Third, in the present study we demonstrated that substitution of LEV-8 by ACR-8 from parasitic nematodes in the *C*. *elegans* L-AChR expressed in *Xenopus* oocyte led to functional rescue of the receptor.

In this context, we reasoned that a model hopping approach utilizing *C*. *elegans lev-8* null mutants could represent a relevant model to investigate the functional role of ACR-8 subunit in parasitic nematodes. Even though we cannot exclude that Hco-ACR-8 could combine with a different set of native muscle AChR subunits, this result strongly supports the hypothesis that ACR-8 from *H*. *contortus* is able to assemble into a functional L-AChR in *C*. *elegans*. In *C*. *elegans*, L-AChR has been proposed as the pharmacological target of Pyr as *unc-63*, *unc-38* and *unc-29* mutants are resistant to 1mM of this drug [40, 41]. Supporting this hypothesis, we showed here that *C*. *elegans* is also resistant to 250μM Pyr, but only partially. This partial Pyr resistance in *lev-8* null mutant could be explained by the expression of functional Cel-L-AChR-2.1/Cel-L-AChR-2.2 in the worms. Indeed, we demonstrated that both of these L-AChR subtypes (that do not include the LEV-8 subunit) are responsive to Pyr when expressed in the *Xenopus* oocyte. An analysis of Pyr sensitivity in a double null mutant, *lev-8*/*acr-8* would be required to test this hypothesis. Surprisingly, *lev-8* null mutants expressing the Hco-ACR-8 were more susceptible to Pyr than wild-type *C*. *elegans*. Because the recombinant *C*. *elegans* L-AChR expressed in *Xenopus* oocyte is less responsive to Pyr than ACh and Lev, we reasoned that the *C*. *elegans* L-AChR in which *lev-8* was substituted by Hco-ACR-8 could be more responsive to Pyr, providing a potential explanation for the increased sensitivity to Pyr in the transgenic worms. However, we found that relative responses to Pyr were similar in both native and composite L-AChRs (S5 Fig). Note that Pyr dose response was not established on *C*. *elegans* L-AChR or the composite receptor due to the small current amplitude. If these results suggest that Hco-ACR-8 does not modulate Pyr sensitivity in the composite receptor, they cannot support a rational explanation for the increased Pyr sensitivity in transgenic *lev-8* null mutant expressing Hco-ACR-8. In that respect, we could only speculate that when expressed in the *C*. *elegans lev-8* null mutant, the parasitic nematode ACR-8 subunit can impact the pharmacological properties or expression of AChR subtypes for which subunit composition remains to be deciphered.

### RNA interference and associated automated real-time larval motility assay: Novel approaches to functionally investigate AChR subunits in parasitic nematodes

Unlike *C*. *elegans*, RNAi approaches in plant and animal parasitic nematode appear capricious and less well controlled [51–53]. In 2015, McCoy *et al*. reported the successful silencing of distinct L-AChR subunit genes *unc-29*, *unc-38* and *unc-63* from the pig parasite *A*. *suum* [54]. However, these experiments performed on adult worms did not allow the identification of any phenotype associated with the AChR subunit gene silencing. More recently, the successful silencing of UNC-38 and UNC-29 associated with an impaired motility was reported in the filarial nematode *Brugia malayi* [18]. For trichostrongylid species such as *H*. *contortus*, *T*. *colubriformis* and *T*. *circumcincta*, the improvement of RNAi efficacy is therefore of critical importance to decipher the pharmacological target of anthelmintics. An important aspect that influences the tractability of the nematode to gene silencing is the ability to take up the siRNA constructs. Therefore, we optimized the gene silencing method in *H*. *contortus*, taking advantage of constitutive feeding in L2 larvae. After 72 h of incubation with siRNA targeting *Hco-unc-63* or *Hco-unc-38*, *H*. *contortus* L2 harbored a strong invalidated phenotype whereas incubation with siRNA targeting *Hco-acr-8* did not induce motility deficiency. These observations are in accordance with the respective phenotype of the *C*. *elegans*, *unc-63*, *unc-38* and *acr-8* null mutants [22, 33]. Note that because of the functional redundancy of the four distinct *unc-29* paralogs of *H*. *contortus* [35], these L-AChR subunits were not investigated by RNAi in the present study.

In order to quantify worm motility reduction resulting from either gene silencing or paralysis associated with drug application, we developed a novel larval migration assay that combines advantages of motility assays performed in filtration plates [55] and real-time video assisted monitoring [16]. This Automated Larval Migration Assay (ALMA) allows real time motility quantification on a large number of worms per set of experiments (up to 10 000) and sees a significant reduction in experimental variability. Therefore, in addition to providing a relevant tool to quantify motility modulation associated with gene silencing, ALMA could also pave the way for a novel drug screening assay.

Taking advantage of this assay, we quantified the phenotypes associated with *Hco-unc-63* and *Hco-unc-38* silencing, providing a first demonstration that L-AChR subunit transcripts from *H*. *contortus* are amenable targets for RNAi experiments.

### New insight into L-AChR subtype diversity in nematodes

Single channel recordings performed on wild-type *C*. *elegans* muscle cells revealed one main L-AChR subtype sensitive to Lev, Pyr and Morantel (Mor) [56]. The systematic analysis of single L-AChR-channel properties from *unc-38*, *unc-63*, *unc-29*, *lev-1*, *lev-8* and *acr-8* null mutants suggested that the main functional L-AChR in wild type worms is made of UNC-38, UNC-63, UNC-29, LEV-1 and LEV-8 [33]. Importantly, whereas in *unc-63*, *unc-29* and *unc-38* mutants Lev-activated muscle currents are abolished, in *lev-1* and *lev-8* mutants they are only reduced [25, 26, 28, 33, 56]. This supports a potential plasticity of *C elegans* L-AChR subtype subunit composition including UNC-63/UNC-38/ UNC-29 as core components in combination with LEV-1 and/or LEV-8. In addition, if single channel recording experiments indicated that Cel-ACR-8 might not be a component of the main L-AChR subtype, analysis of the double null mutant *lev-8*/*acr-8* strongly suggested that ACR-8 is able to replace LEV-8 in the heteropentamer when this subunit is absent [33]. In accordance with this observation, in the present work we demonstrated that Cel-ACR-8 is able to associate with UNC-63, UNC-38, UNC-29 and LEV-1 to form functional L-AChRs when expressed in the *Xenopus* oocytes. These results highlight the need to further explore the diversity of *C*. *elegans* L-AChR subtypes as they could contribute to a better understanding of the synapse biology but also might be used as reference for comparative studies with parasitic nematode L-AChRs lacking LEV-8 and/or LEV-1 subunits. In comparison with wild-type *C*. *elegans*, electrophysiological experiments revealed an even greater functional and pharmacological diversity of L-AChRs in parasitic nematode species. For example, in *O*. *dentatum* (Clade V) single channel recordings revealed up to four distinct L-AChR conductance states [57]. The diversity of muscle AChRs has been investigated in *H*. *contortus* and *O*. *dentatum* [34, 36]. For both species, using the *Xenopus* oocyte as an expression system, combination of UNC-38, UNC-63, UNC-29 and ACR-8 subunits led to the functional expression of L-AChRs preferentially activated by Lev (Hco-L-AChR-1 for *H*. *contortus*, Ode (29-63-8-38) for *O*. *dentatum*) whereas the same subunit combination lacking ACR-8 led to the functional expression of another AChR subtypes preferentially activated by Pyr (Hco-L-AChR-2 for *H*. *contortus*, Ode (29-63-38) for *O*. *dentatum*). These recombinant experiments reinforce the potential for distinct subtypes of L-AChR assembled from distinct subunit combinations in animal parasitic nematodes.

In accordance with this, the present work demonstrated that silencing of the *Hco-acr-8* gene reduced Lev sensitivity of *H*. *contortus* L2 without impacting their Pyr sensitivity. This observation supports the notion of distinct subtypes of muscle L-AChR and the hypothesis that Lev and Pyr act on distinct AChR subtypes in *H*. *contortus*. Recently, we described a novel muscle AChR subtype made of ACR-26/ACR-27 subunits that is specific to parasitic nematodes. The co-expression of ACR-26/ACR-27 from *H*. *contortus* in *C*. *elegans* N2 conferred an increased sensitivity to both Pyr and Mor in the transgenic worms [38]. Thus, we could speculate that the absence of Pyr sensitivity in the face of *Hco-acr-8* gene silencing is underpinned by the presence of alternative functional Pyr-sensitive-AChRs which might include the putative Hco-L-AChR-2 and/or Hco-26/27 receptors. In conclusion, whereas *C*. *elegans* remains a highly valuable model for deciphering the pharmacological targets of cholinergic anthelmintics, the differences between the prototypic L-AChR of *C*. *elegans* and its counterparts in parasitic nematodes highlight the need to further explore AChRs in target parasitic species. This is particularly pertinent in view of the pivotal role these targets play in drug treatment and resistance. In the present study, we used a set of complementary approaches to investigate specific functions of AChR subunits from nematodes and paved the way for a better understanding of anthelmintic mode of action in a wide range of species. Such information will be of major importance for the rational development of drug combination as well as the improvement of target-based drug screening.

## Methods

### Ethics statement

All animal care and experimental procedures were conducted in strict accordance with the European guidelines for the care and use of laboratory animals and were approved by French ministry of teaching and research and the regional Val de Loire ethics committee (no 19) as a protocol registered under the number 00219.02 in the experimental installations (n° agreement: C371753). For the purpose of this study, three-month-old sheep were infected with 6000 infective larvae (L3) from *Haemonchus contortus* to extract nematode's eggs from fresh fecal material and collect adult worms after necropsy. For the three other animal parasitic nematode species used in the present study (i.e. *Ascaris suum*, *Dirofilaria immitis* and *Oesophagostomum dentatum*), adult worms were obtained from already-existing collections.

### Nematodes

*Haemonchus contortus* experiments were performed on the Weybridge isolate as previously described [58]. *Dirofilaria immitis* adult male worms were supplied by the Filarial Research Reagent Resource Center, University of Georgia [59]. *Meloidogyne incognita* nematodes (Morelos strain) were cultured on greenhouse-grown tomato plants at the French National Institute for Agricultural Research (UMR 1355 ISA, Sophia-Antipolis) and collected as described previously [60]. The adult worms of *Ascaris suum* and *Oesophagostomum dentatum* levamisole-sensitive isolate (SENS) were obtained from the French National Institute for Agricultural Research; UMR 1282 ISP (Nouzilly) nematode collection [61]. *Caenorhabditis elegans* experiments were carried out on the Bristol N2 wild-type and *lev-8(ok1519)* strains obtained from the *Caenorhabditis* Genetics Center (CGC).

### cDNA synthesis

Total RNA was prepared from the distinct nematode species using: 10 adult males or 50μL of pelleted eggs, L2 or L3 larvae of *H*. *contortus*, cross-section (5mm thick) from the mid body region of an individual adult worm of *Ascaris suum*, 5 adult males of *D*. *immitis*, 50μl of pelleted J2 stages of *M*. *incognita*, 10 adult males of *O*. *dentatum* or 100μL of pelleted mixed stages of *C*. *elegans* respectively. Frozen samples were ground in liquid nitrogen and homogenized in Trizol reagent (Invitrogen, Carlsbad, CA, USA) and total RNA was isolated according to the manufacturer’s recommendations. RNA pellets were dissolved in 25 μL of RNA secure resuspension solution (Ambion, Austin, TX, USA) and DNase-treated using the TURBO DNA-free kit (Ambion). RNA concentrations were measured using a Nanodrop spectrophotometer (Thermo Scientific, Waltham, MA, USA). First-strand cDNA synthesis was performed on 1μg of total RNA using the superscript III reverse transcriptase (Invitrogen, Carlsbad, CA, USA) according to the manufacturer’s recommendations.

### Cloning of complete coding cDNA sequences of *acr-8* from *A*. *suum*, *C*. *elegans*, *D*. *immitis* and *M*. *incognita*

Complete coding sequences corresponding to *Asu-acr-8*, *Cel-acr-8*, *Dim-acr-8 and Min-acr-8*, were obtained using first-strand cDNA as PCR template. Amplifications were performed with the Phusion High fidelity Polymerase (New England Biolabs) with a forward primer including the first ATG codon in combination with a reverse primer including the first stop codon. Amplicons were inserted in the transcription vector pTB207 [27] using the In-Fusion HD cloning kit (Clontech). Primer sequences are reported in S2 Table. The novel complete coding sequences of *Asu-acr-8*, *Dim-acr-8* and *Min-acr-8* were deposited to GenBank under the accession numbers: KY654347, KY654349 and KY654350, respectively.

### Sequence analysis

Database searches were performed with the tBLASTn or BLASTP algorithms [62]. Deduced protein sequences were aligned using the MUSCLE software [63]. Amino-acids homologies and identities were defined using the EMBOSS Needle program available at EMBL-EBI (http://www.ebi.ac.uk/Tools/psa/emboss_needle/). Signal peptide predictions were performed using the SignalP 4.1 server [64] and membrane-spanning regions were predicted using the SMART server [65]. Phylogenetic analyses were performed on coding sequences predicted from genomic data available in databases or cloned cDNA sequences when available. Sequences were aligned as codons using the MAFFT plugin (v1.3.3) of Geneious (v7.1.2, Biomatters Ltd) [66]. Regions corresponding to the signal peptide and the intracellular loop between TM3 and TM4 that could not be aligned unambiguously were removed. Maximal likelihood phylogeny reconstruction was performed using PhyML v20120412 (https://github.com/stephaneguindon/phyml-downloads/releases) as previously described in Duguet *et al*. 2016 [35]. Standard nomenclature of nematode species: Aca: *Ancylostoma caninum*; Ace: *Ancylostoma ceylanicum*; Acs: *Angiostrongylus costaricensis*; Asu: *Ascaris suum*; Avi: *Acanthocheilonema viteae*; Bma: *Brugia malayi*; Bxy: *Bursaphelenchus xylophilus*; Cbr: *Caenorhabditis briggsae*; Cel: *Caenorhabditis elegans*; Cjp: *Caenorhabditis japonica*; Cre: *Caenorhabditis remanei*; Dim: *Dirofilaria immitis*; Dme: *Dracunculus medinensis*; Dvi: *Dictyocaulus viviparus*; Eel: *Elaeophora elaphi*; Eve: *Enterobius vermicularis*; Gpu: *Gongylonema pulchrum*; Hba: *Heterorhabditis bacteriophora*; Hbk: *Heligmosomoides polygyrus*; Hco: *Haemonchus contortus*; Llo: *Loa loa*; Mfl: *Meloidogyne floridensis*; Mha: *Meloidogyne hapla*; Min: *Meloidogyne incognita*; Nam: *Necator americanus*; Nbr: *Nippostrongylus brasiliensis*; Ode: *Oesophagostomum dentatum*; Ovo: *Onchocerca volvulus*; Ppa: *Pristionchus pacificus*; Ptr: *Parastrongyloides trichosuri*; Rcu: *Romanomermis culicivorax*; Rhb: *Rhabditophanes* kr3021; Smu: *Syphacia muris*; Spa: *Strongyloides papillosus*; Sra: *Strongyloides ratti*; Sst: *Strongyloides stercoralis*; Svz: *Strongyloides venezuelensis*; Tca: *Toxocara canis*; Tci: *Teladorsagia circumcincta*; Tco: *Trichostrongylus colubriformis*; Tmu: *Trichuris muris*; Tna: *Trichinella nativa*; Tsp: *Trichinella spiralis*; Tsu: *Trichuris suis*; Ttr: *Trichuris trichiura*; Tzc: *Thelazia callipaeda*; Wba: *Wuchereria bancrofti*.

### Expression of *Hco-acr-8*, *Hco-unc-63* and *Hco-unc-38* through the *H*. *contortus* free-living stages

PCR was carried out on *H*. *contortus* first strand cDNA prepared from eggs, L2 and L3 larvae. PCR reactions were carried out in a final volume of 20μl, containing 100ng of first strand cDNA, 1 unit of GoTaq polymerase (Promega), 0.25mM dNTPs each and 0.3μM of each primer. The reaction mixture was denatured by heating to 94°C for 5 min, followed by 34 cycles of 94°C for 45 sec, 56°C for 45 sec, 72°C for 45 sec. A final extension step was performed at 72°C during 5 min. *H*. *contortus* GAPDH (HM145749) was used as the reference transcript. Primer sequences are provided in S2 Table.

### Electrophysiology experiments

*A*. *suum*, *C*. *elegans*, *D*. *immitis*, and *M*. *incognita acr-8* cDNAs were PCR-amplified to be sub-cloned into the expression vector pTB207 that is suitable for *in vitro* transcription [27]. Subcloning experiments of *H*. *contortus* and *O*. *dentatum acr-8* cDNA in pTB207 have been previously reported [34, 36]. *C*. *elegans unc-63*, *unc-38*, *unc-29*, *lev-8* and *lev-1* cDNA subcloned in pTB207 were kindly provided by Thomas Boulin [27]. The plasmids were linearized with the *NheI* restriction enzyme (Fermantas) and used as templates for cRNA synthesis using the T7 mMessage mMachine kit (Ambion). Defolliculated *Xenopus laevis* oocytes were obtained from Ecocyte (Germany). The oocytes were injected in the animal pole with a total volume of 36nL of cRNA mix containing 50ng/μL of each cRNA in RNase-free water using the Drummond Nanoject II microinjector. Microinjected oocytes were kept at 20°C in incubation medium (100mM NaCl, 2mM KCl 2, 1.8mM CaCl_2_.2H2O, 1mM MgCl2.6H2O, 5mM HEPES, 2.5mM C_3_H_3_NaO_3_, pH 7.5, supplemented with penicillin 100 U/mL and streptomycin 100μg/mL) for 4 days to allow the receptors expression. Two-electrode voltage-clamp recordings were carried out as previously described [38].

### Expression of GFP driven by *Hco-acr-8* promoter

A 2,9 kb region upstream the initiation codon of *Hco-acr-8* was PCR amplified on genomic DNA prepared from adult males of *H*. *contortus* using the NucleoSpin Tissue kit (Macherey-Nagel, Duren, Germany) following manufacturer recommendations. Primers were designed upon scaffold 5709 sequence available in *H*. *contortus* PRJEB506 Wormbase parasite database. Primer sequences are provided in S2 Table. The amplicon was subcloned upstream of the GFP coding sequence in the pPD95.75 (Addgene) expression vector and micro-injected into *C*. *elegans* N2 gonad (50 ng μl^-1^). Expression of GFP was monitored on fixed wormed from the F1 progeny by fluorescence microscopy.

### *C*. *elegans* rescue experiments-transgenics

*Hco-acr-8* coding sequence was sub-cloned into the pPD96.52 (Addgene) vector containing a myosin promoter *Pmyo3*, using the In-FusionHD cloning kit (Clontech). Primer sequences are listed in S2 Table. *C*. *elegans lev-8 (ok1519)* were injected with 30ng μl^-1^ of the resulting plasmid to drive the expression of *H*. *contortus acr-8* in the body muscle cells. Transformed worms were identified by co-injecting pPD118.33 (*Pmyo-2*::*gfp*) plasmid at 50ng μl^-1^ (Fire lab vector kit), which drives expression of green fluorescent protein (GFP) from the pharyngeal muscle promoter *Pmyo-2*. The co-injected *gfp* transformation marker forms an extra-chromosomal array with the plasmids carrying the gene sequence and thus worms with fluorescent green pharyngeal muscle can be identified as carrying the plasmid of interest. For all the experiments, two independently transformed stable lines of transgenic *C*. *elegans* were assayed.

### *C*. *elegans* thrashing assays

Experiments were performed on age synchronized worms by picking L4 stage a day before the assay. The next day, one-day old adult hermaphrodite *C*. *elegans* were picked into 900 μL of M9 buffer containing 0.1% BSA in a 12 well plate. The worms were left to settle for 10 min. The number of thrashes was counted for 1 min. Then 100 μL of either M9 (control), Lev or Pyr solution was added to the wells to give a final concentration of 10, 25, 50, 100 or 150μM Lev or Pyr. The number of thrashes was counted after 10, 20, 30 and 40 min (and 60 min for some concentrations) of exposure to Lev. The data were plotted as a mean ± standard error of the mean.

### Ingestion and RNAi experiments

L2 larvae were obtained from *in vitro* culture of eggs using the standard procedure described by Rossanigo and Gruner (1991) [67]. Approximately 2000–3000 eggs/mL were cultured horizontally in tissue culture flasks at 20°C in a nutritive medium (0.1mL per mL of culture of 1X Earle's balanced salt solution (Sigma-Aldrich) and 0.5% (w/v) of yeast extract). The experimental procedures for soaking assays were performed on 7500 (2 days old) L2 larvae in a final volume of 600μL in 5mL tubes. 1μM non-target (*gfp*) siRNA labeled with the Alexa 594 (Eurogentec, Belgium) was added to the culture medium. Each tube was incubated horizontally at 20°C on a rocking table. After 2 hours of incubation, L2 larvae were washed three times with tap water and ingestion was monitored under fluorescent microscopy. The absence of toxicity was confirmed by comparing the viability of dsRNA treated vs untreated L2 larvae after 72h of incubation. Double strand RNA targeting specifically *Hco-unc-63*, *Hco-unc-38* and *Hco-acr-8* respectively were synthesized by Eurogentec (Belgium). Soaking of larvae in 1μM dsRNA was performed for all the experiments as described for ingestion assays and putative phenotypes were visually checked on a daily basis (up to 96h). The RNAi experiments were performed at least three times in independent replicates. The custom siRNA duplex sequences are provided in S2 Table. Control siRNA duplex targeting *gfp* were purchased from Eurogentec.

### Automated larval migration assay

Larval motility was estimated by measuring *H*. *contortus* L2 auto-fluorescence using a Quanta Master spectrofluorometer (Horiba PTI, NJ, USA). Motility assays were performed using 7500 *H*. *contortus* L2 larvae. Worms were transferred into a 5mL glass tube and left for 15 min to concentrate by gravity. The supernatant was removed and replace by 2 mL of tap water or anthelmintic solution. After 5 min., the tube was inverted on a 20μm sieve. After a stabilization time of 60 sec, the fluorescence accumulation (correlated to the number of larvae migrating through the sieve) was measured during 25 minutes. The recording rates were either one measure /sec (Fig 5E, S8D Fig) or one measure every 4 sec using a four positions sample holder (Horiba PTI, NJ, USA) allowing the synchronized recording of 4 distinct samples (Figs 5A, 5C, 6A and 6B, S8E Fig). Each set of experiment was performed in triplicate. The final migration percentage relative to control was estimated using the mean data of the ten last fluorescence measures.

## Materials

Acetylcholine chloride (ACh), (-)-tetramisole hydrochloride (levamisole), pyrantel citrate, were purchased from Sigma-Aldrich.

### Accession numbers

The accession numbers of annotated cDNA sequences mentioned in this article are:

***Ascaris suum*:** ACR-8 KY654347; ***Caenorhabditis elegans*:** ACR-8 NP_509745; ACR-12 NP_510262; ACR-14 NP_495716; ACR-16 NP_505207; LEV-1 NP_001255705; LEV-8 NP_509932; UNC-29 NP_492399; UNC-38 NP_491472; UNC-63 NP_491533. ***Haemonchus contortus*:** ACR-8 EU006785; UNC-38 GU060984; UNC-63 GU060985; RIC3.1 HQ116823; UNC-74 HQ116821; UNC-50 HQ116822; GAPDH HM145749; ***Oesophagostomum dentatum***: ACR-8 JX429921; ***Teladorsagia circumcincta*:** ACR-8 HQ215517; ***Trichostrongylus colubriformis*:** ACR-8 HQ215518; ***Meloidogyne incognita*:** ACR-8 KY654350. ***Dirofilaria immitis*:** ACR-8 KY654349.

## Supporting information

S1 Fig Maximum likelihood tree showing relationships of parasitic nematodes acetylcholine receptor (AChR) subunits from the ACR-8 group with *C*. *elegans* AChR subunits involved in its L-AChR composition: UNC-38, UNC-63, LEV-1, UNC-29 and LEV-8. Tree was built upon an alignment of AChR subunit sequences excluding the predicted signal peptide and rooted with the *C*. *elegans* ACR-16 subunit sequence. Branch labels correspond to SH values. Scale bar represents the number of substitution per site. *C*. *elegans* AChR subunit groups were named as proposed by Mongan *et al*. [69]. The three-letter prefix in AChR subunit gene names, Ace, Asu, Bxy, Cel, Dim, Min, Ode, Tca, Tna and Ttr refers to *Ancylostoma ceylanicum*, *Ascaris suum*, *Bursaphelenchus xylophilus*, *Caenorhabditis elegans*, *Dirofilaria immitis*, *Haemonchus contortus*, *Meloidogyne incognita*, *Oesophagostomum dentatum*, *Toxocara canis*, *Trichinella nativa* and *Trichuris trichiura*, respectively. ACR-8-related sequences are highlighted in purple, ACR-8 orthologs are highlighted in blue, LEV-8 orthologs are highlighted in red. The node corresponding to the putative duplication event is indicated by a green star.
(TIF)

S2 Fig Alignments of ACR-8 AChR subunit sequences from parasitic nematodes with ACR-8 and LEV-8 AChR subunit sequences from *C*. *elegans*. The *acr-8* deduced amino-acid sequences from *C*. *elegans*, *H*. *contortus*, *O*. *dentatum*, *A*. *suum*, *D*. *immitis* and *M*. *incognita* were aligned with ACR-8 and LEV-8 sequences from *C*. *elegans* using the MUSCLE algorithm [58]. Predicted signal peptide sequences are shaded in grey. Amino acids conserved between by ACR-8 and LEV-8 sequences are highlighted in dark blue. Amino acids specific to ACR-8 sequences are highlighted in light blue. Amino acids specifically shared by ACR-8 homologs from parasitic species are highlighted in green. Amino acids conserved between Cel-LEV-8 and parasitic nematode ACR-8 sequences -but not *C*. *elegans* ACR-8- are highlighted in red. The Cys-loop, the four transmembrane regions (TM1-TM4) and the primary agonist binding site (YxGCC) are noted above the sequences.
(TIF)

S3 Fig Representative recording traces from single *Xenopus* oocytes expressing *C*. *elegans* L-AChR subtypes or composite L-AChRs containing distinct parasite AChR subunits. **A.** Representative recording traces of currents elicited by 100μM acetylcholine (ACh) or 100μM levamisole (Lev) application on the *C*. *elegans* L-AChR (UNC-29, UNC-38, UNC-63, LEV-1, LEV-8).
**B-F.** 100μM ACh or 100μM Lev elicited currents on composite *C*. *elegans* L-AChRs including Cel-UNC-29, Cel-UNC-38, Cel-UNC-63, Cel-LEV-1 and the ACR-8 subunit from a parasitic nematode species: *H*. *contortus* ACR-8 (**B**), *O*. *dentatum* ACR-8 (**C**), *A*. *suum* ACR-8 (**D**), *D*. *immitis* ACR-8 (**E**) and *M*. *incognita* ACR-8 (**F**).
**G-H.** 100μM ACh or 100μM Lev elicited currents on composite *C*. *elegans* L-AChRs with Hco-UNC-38 replacing Cel-UNC-38 (**G**) or Hco-UNC-63 replacing Cel-UNC-63 (**H**).
**I-J.** Representative recording traces of currents elicited by 100μM acetylcholine (ACh) or 100μM levamisole (Lev) application on the *C*. *elegans* L-AChR-2.1 (UNC-29, UNC-38, UNC-63, LEV-1, ACR-8), (**I**) and L-AChR-2.1 (UNC-29, UNC-38, UNC-63, ACR-8), (**J**). The bars indicate the time period of the agonist application.
(TIF)

S4 Fig Representative recording traces from a single *Xenopus* oocyte expressing Hco-UNC-63/Hco-UNC-38/Hco-UNC-29.1/Cel-LEV-8 (**A**), Hco-UNC-63/Hco-UNC-38/Hco-UNC-29.1/Cel-ACR-8 (**B**) or Hco-UNC-63/Hco-UNC-38/Hco-UNC-29.1 (**C**) challenged with 100μM ACh or 100μM Lev. The bars indicate the time period of the agonist application. **D**) Scatter plot (mean ± SEM) of currents elicited by 100μM ACh or 100μM Lev on Hco-UNC-63/Hco-UNC-38/Hco-UNC-29.1/Cel-LEV-8; Hco-UNC-63/Hco-UNC-38/Hco-UNC-29.1/Cel-ACR-8 or Hco-UNC-63/Hco-UNC-38/Hco-UNC-29.1, respectively. Number of oocytes is reported on the graph.
(TIF)

S5 Fig Effects of 100μM Pyr application on *Xenopus* oocytes expressing *C*. *elegans* L-AChR subtypes or composite L-AChRs. Representative recording traces of currents elicited by 100μM Pyr application on the Cel-L-AChR-2.1 (**A**), Cel-L-AChR-2.2 (**B**), Hco-L-ACHR-1 with Cel-ACR-8 replacing Hco-ACR-8 (**C**), Cel-L-AChR (**D**), Hco-L-AChR-1 (**E**), Cel-L-AChR with Hco-ACR-8 replacing Ce-LEV-8 (**F**).
The bars indicate the time period of the agonist application. I_max_ Pyr values (% of 100μM ACh response) are reported for each receptor above their respective representative recording traces.
(TIF)

S6 Fig Expression of *H*. *contortus* ACR-8 promoter fused to GFP or Hco-ACR-8 subunit in *C*. *elegans*. **A.** Expression patterns in *C*. *elegans* (N2) of GFP and mCherry driven by the *H*. *contortus acr-8* and *C*. *elegans myo-3* promoters respectively. **B.** Thrashing rate in M9 medium of *C*. *elegans* N2, *lev-8(ok1519)* and transgenic *lev-8(ok1519)* expressing Hco-ACR-8.A thrashing rate was established for wild type N2, *lev-8(ok1519)* and two lines of transgenic *lev-8(ok1519); Pmyo-3*::*hco acr-8 C*. *elegans* after 10, 20, 30 and 40 min in M9 medium. Data are the mean ± SEM.
(TIF)

S7 Fig Effects levamisole or pyrantel on the thrashing rate of *lev-8(ok1519)* expressing *H*. *contortus acr-8*. A thrashing rate was established for wild type N2, *lev-8(ok1519)* and two lines of transgenic *lev-8(ok1519); Pmyo-3*::*hco acr-8 C*. *elegans* after 10, 20, 30 and 40 min of exposure to Lev 10μM (**A**), 50μM (**B**), 100μM (**C**), 200μM (**D**) or Pyr 10μM (**E**), 25μM (**F**), 50μM (**G**) and 100μM (**H**) respectively. Basal thrashing rate was established after 10 min acclimatisation in M9 buffer. Data are the mean ± SEM of n ≥8, ****p<0.0001, ***p<0.001, **p<0.01 and *p<0.05, one way ANOVA with Bonferroni post-hoc test between basal and after drug treatment thrashing rate for the same strain. In black: wild-type N2 strain, in dark red: *lev-8 (ok1519)*, in blue: *cePmyo-3*::*hco-acr-8* l.1, in purple: *cePmyo-3*::*hco-acr-8* l.2.
(TIF)

S8 Fig *Haemonchus contortus* L2 developmental stage is relevant for L-AChR subunits investigation using RNAi associated with an automated larval migration assay. **A.** Expression of *Hco-unc-29*.*1*, *Hco-acr-8*, *Hco-unc-63 and Hco-unc-38* in the free-living stages of *Haemonchus contortus*. Transcription of *Hco-unc-29*.*1*, *Hco-acr-8*, *Hco-unc-63 and Hco-unc-38* throughout the free-living stages of *H*. *contortus* was investigated by RT-PCR; ω: embryonated egg; L2: second stage larvae; L3: third stage larvae. Integrity of cDNA preparations was verified by PCR using primers designed to amplify a fragment of the *H*. *contortus gapdh* cDNA.
**B-C.** Monitoring of labelled siRNA ingestion by *Haemonchus contortus* second stage larvae. *H*. *contortus* L2 larvae incubated during 2 hours in a culture medium containing 1μM of non-specific siRNA (targeting *gfp*) labelled with Alexa 594 (**B**). Scale bar: 75μm. Negative control without fluorochrome added in the culture medium(**C**). Scale bar: 35μm.
**D.** *H*. *contortus* L2 larvae migration assay using auto-fluorescence quantification. Correlation between the fluorescence counting (counts/sec) and the number of L2 larvae that migrated through the 30μM sieve during 25min. Migration assays were performed using 1000, 2500, 5000, 7500 and 10000 L2 larvae respectively. Each data point represents mean± SE of three independent runs.
**E.** Non-target siRNA does not modulate motility / levamisole sensitivity of *H*. *contortus* L2 larvae. The automated larval migration assay (ALMA) was used to determine the putative impact of siRNA targeting *gfp* on migration or Lev sensitivity (0.3μM) of *H*. *contortus* L2 larvae. **A.** Representative recording traces of the real-time fluorescence counting relative to the L2 migration during 25min. Each trace corresponds to the mean data from 3 runs performed with 7500 L2 larvae. The control corresponds to untreated L2 larvae.
(TIF)

S1 Table Identification of Cel-*acr-8*and Cel *lev-8* homologs in nematode genomic data available in Wormbase-parasite databank. Deduced amino-acid sequences from Cel-ACR-8 (Genbank accession number: JF416644.1) and Cel-LEV-8 (Genbank accession number: NM_077531.4) were used to perform a tBlastn search against a set of genomic databanks from nematodes available in *WormBase Parasite* (https://parasite.wormbase.org/). Nematode Clades as determined by Blaxter *et al*. [68].When available, full length cDNA sequences are indicated in bold: GB refers to Genbank accession number whereas WB refers to Worm base accession number. Life style abbreviations: VP: vertebrate parasite; IP: Insect parasite; FL: free-living.
(DOCX)

S2 Table Sequences of primers and ds-siRNA. (DOCX)

S1 Video *H*. *contortus* L2 larvae pharyngeal pumping in culture medium. (MPG)

S2 Video *H*. *contortus* L2 larvae in culture medium without siRNA (control). (MPG)

S3 Video *H*. *contortus* L2 larvae exposed to 1μM siRNA targeting *Hco-unc-38* after 72h. (MPG)

S4 Video *H*. *contortus* L2 larvae exposed to 1μM siRNA targeting *Hco-unc-63* after 72h. (MPG)

S5 Video *H*. *contortus* L2 larvae exposed to 1μM siRNA targeting *Hco-acr-8* after 72h. (MPG)
