# Supplementary figures and images for: Deciphering the molecular determinants of cholinergic anthelmintic sensitivity in nematodes: When novel functional validation approaches highlight major differences between the model Caenorhabditis elegans and parasitic species
Source: PLoS Pathog. 2018 May 2;14(5):e1006996. doi: 10.1371/journal.ppat.1006996 (PMC5931475; doi:10.1371/journal.ppat.1006996)

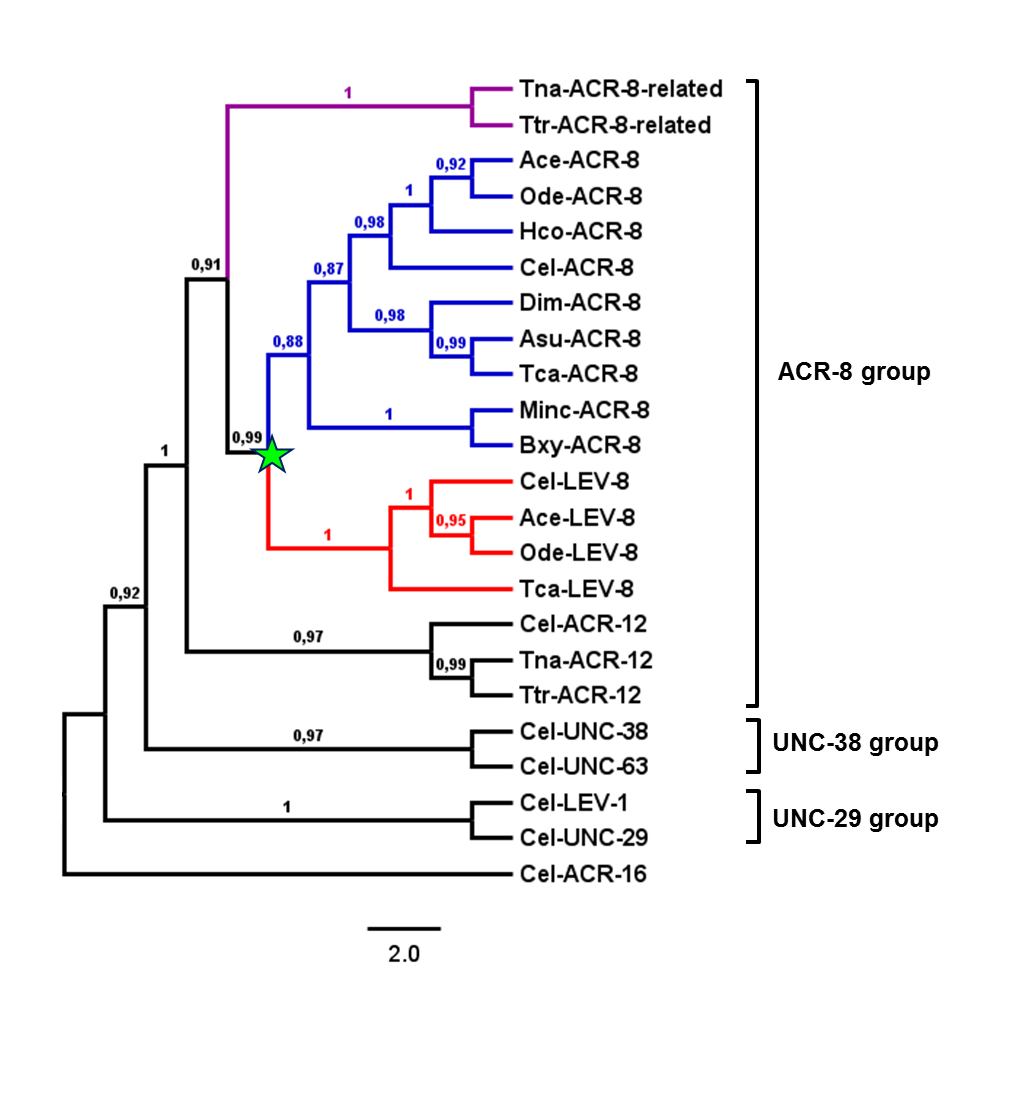

Supplement: S1 Fig — Tree was built upon an alignment of AChR subunit sequences excluding the predicted signal peptide and rooted with the C. elegans ACR-16 subunit sequence. Branch labels correspond to SH values. Scale bar represents the number of substitution per site. C. elegans AChR subunit groups were named as proposed by Mongan et al. [69]. The three-letter prefix in AChR subunit gene names, Ace, Asu, Bxy, Cel, Dim, Min, Ode, Tca, Tna and Ttr refers to Ancylostoma ceylanicum, Ascaris suum, Bursaphelenchus xylophilus, Caenorhabditis elegans, Dirofilaria immitis, Haemonchus contortus, Meloidogyne incognita, Oesophagostomum dentatum, Toxocara canis, Trichinella nativa and Trichuris trichiura, respectively. ACR-8-related sequences are highlighted in purple, ACR-8 orthologs are highlighted in blue, LEV-8 orthologs are highlighted in red. The node corresponding to the putative duplication event is indicated by a green star. (TIF) [file ppat.1006996.s001.tif]

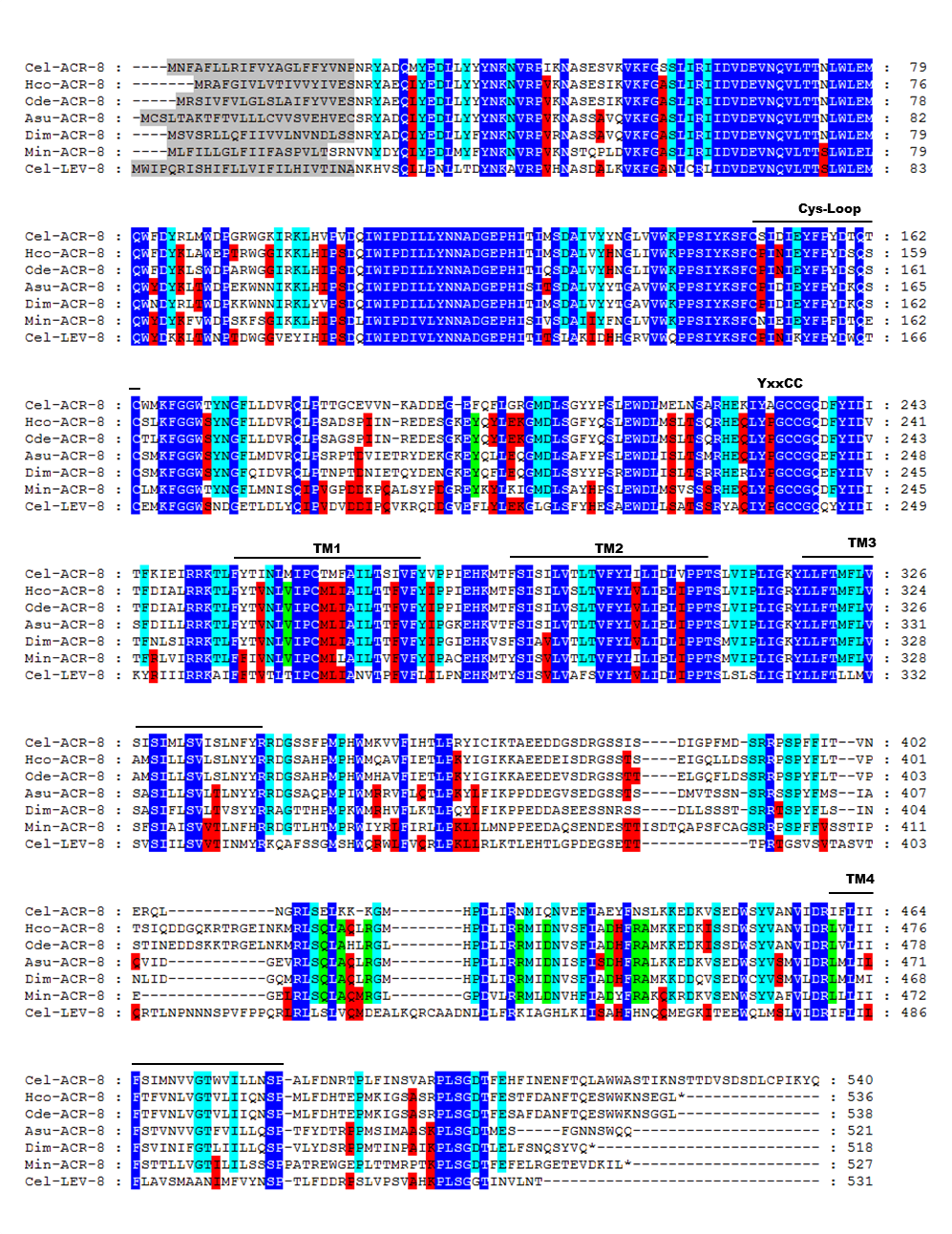

Supplement: S2 Fig — The acr-8 deduced amino-acid sequences from C. elegans, H. contortus, O. dentatum, A. suum, D. immitis and M. incognita were aligned with ACR-8 and LEV-8 sequences from C. elegans using the MUSCLE algorithm [58]. Predicted signal peptide sequences are shaded in grey. Amino acids conserved between by ACR-8 and LEV-8 sequences are highlighted in dark blue. Amino acids specific to ACR-8 sequences are highlighted in light blue. Amino acids specifically shared by ACR-8 homologs from parasitic species are highlighted in green. Amino acids conserved between Cel-LEV-8 and parasitic nematode ACR-8 sequences -but not C. elegans ACR-8- are highlighted in red. The Cys-loop, the four transmembrane regions (TM1-TM4) and the primary agonist binding site (YxGCC) are noted above the sequences. (TIF) [file ppat.1006996.s002.tif]

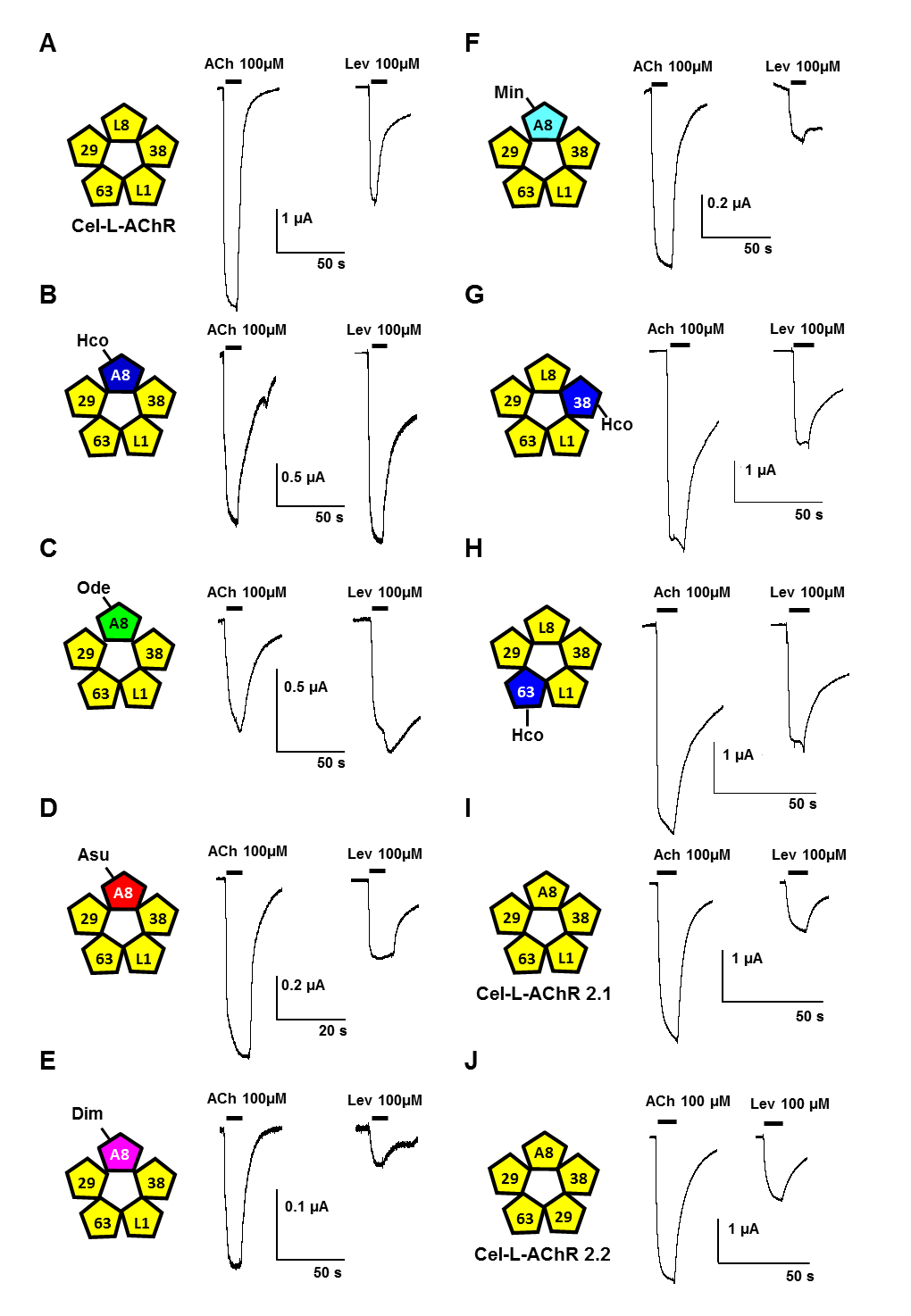

Supplement: S3 Fig — A. Representative recording traces of currents elicited by 100μM acetylcholine (ACh) or 100μM levamisole (Lev) application on the C. elegans L-AChR (UNC-29, UNC-38, UNC-63, LEV-1, LEV-8). B-F. 100μM ACh or 100μM Lev elicited currents on composite C. elegans L-AChRs including Cel-UNC-29, Cel-UNC-38, Cel-UNC-63, Cel-LEV-1 and the ACR-8 subunit from a parasitic nematode species: H. contortus ACR-8 (B), O. dentatum ACR-8 (C), A. suum ACR-8 (D), D. immitis ACR-8 (E) and M. incognita ACR-8 (F). G-H. 100μM ACh or 100μM Lev elicited currents on composite C. elegans L-AChRs with Hco-UNC-38 replacing Cel-UNC-38 (G) or Hco-UNC-63 replacing Cel-UNC-63 (H). I-J. Representative recording traces of currents elicited by 100μM acetylcholine (ACh) or 100μM levamisole (Lev) application on the C. elegans L-AChR-2.1 (UNC-29, UNC-38, UNC-63, LEV-1, ACR-8), (I) and L-AChR-2.1 (UNC-29, UNC-38, UNC-63, ACR-8), (J). The bars indicate the time period of the agonist application. (TIF) [file ppat.1006996.s003.tif]

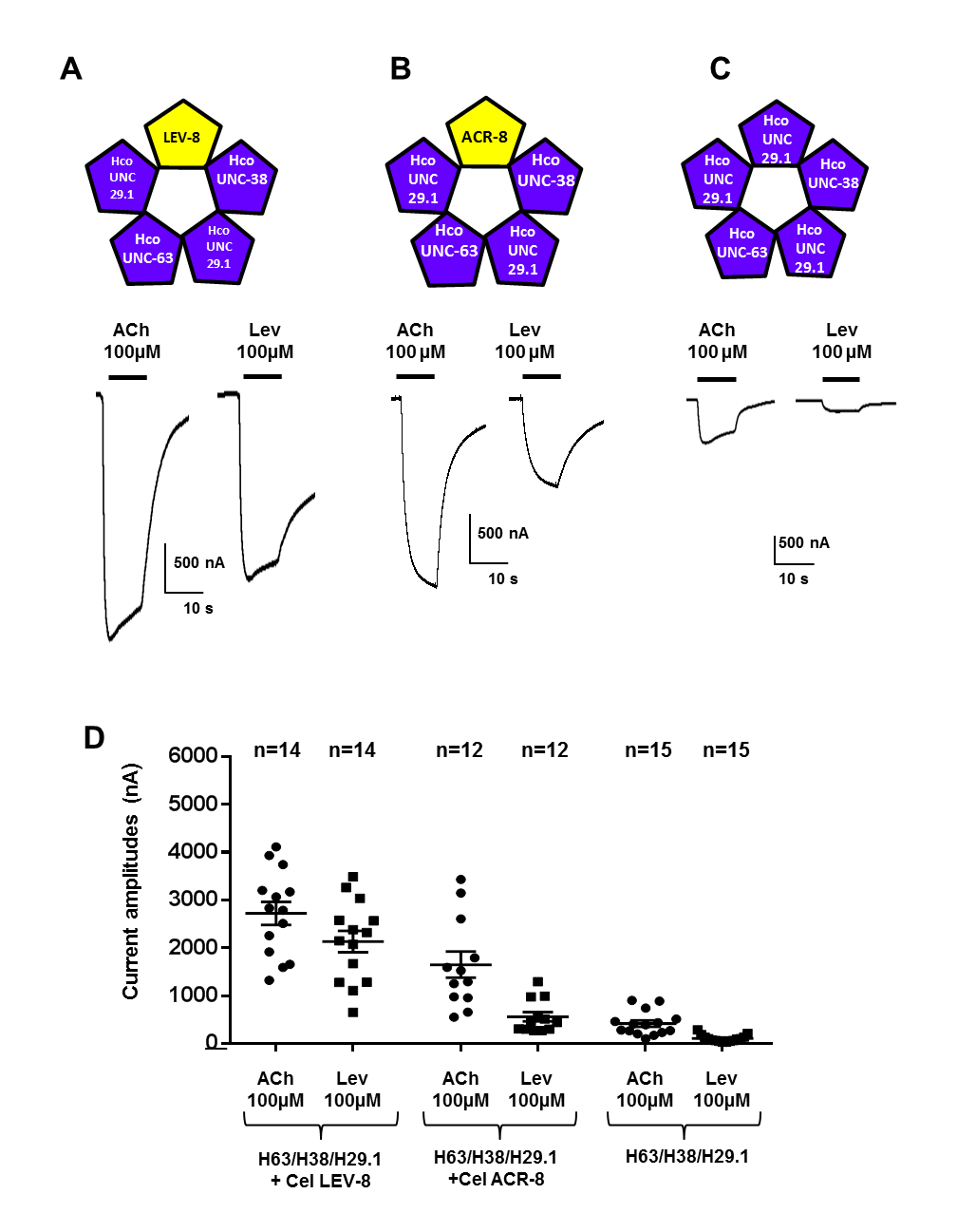

Supplement: S4 Fig — Representative recording traces from a single Xenopus oocyte expressing Hco-UNC-63/Hco-UNC-38/Hco-UNC-29.1/Cel-LEV-8 (A), Hco-UNC-63/Hco-UNC-38/Hco-UNC-29.1/Cel-ACR-8 (B) or Hco-UNC-63/Hco-UNC-38/Hco-UNC-29.1 (C) challenged with 100μM ACh or 100μM Lev. The bars indicate the time period of the agonist application. D) Scatter plot (mean ± SEM) of currents elicited by 100μM ACh or 100μM Lev on Hco-UNC-63/Hco-UNC-38/Hco-UNC-29.1/Cel-LEV-8; Hco-UNC-63/Hco-UNC-38/Hco-UNC-29.1/Cel-ACR-8 or Hco-UNC-63/Hco-UNC-38/Hco-UNC-29.1, respectively. Number of oocytes is reported on the graph. (TIF) [file ppat.1006996.s004.tif]

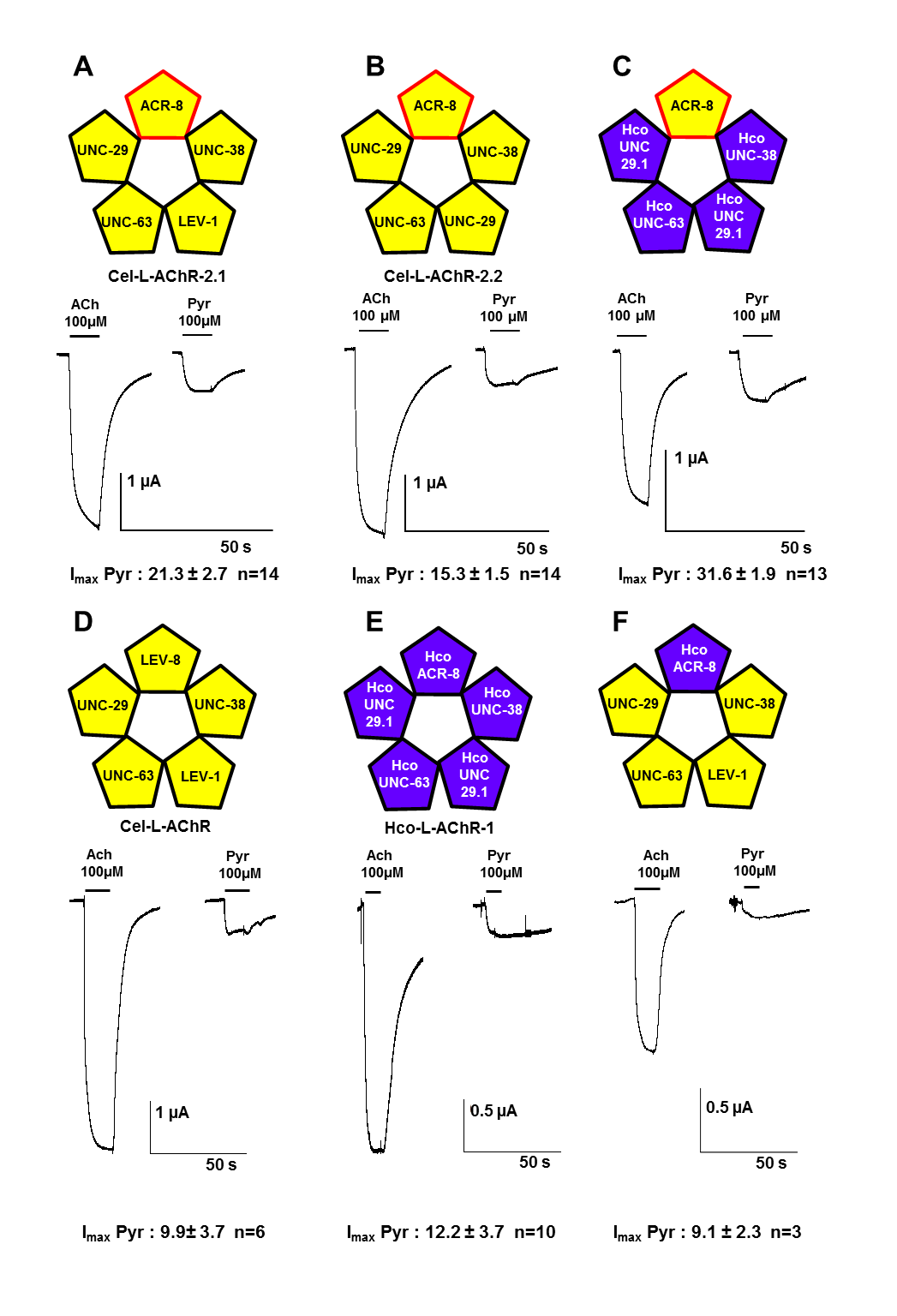

Supplement: S5 Fig — Representative recording traces of currents elicited by 100μM Pyr application on the Cel-L-AChR-2.1 (A), Cel-L-AChR-2.2 (B), Hco-L-ACHR-1 with Cel-ACR-8 replacing Hco-ACR-8 (C), Cel-L-AChR (D), Hco-L-AChR-1 (E), Cel-L-AChR with Hco-ACR-8 replacing Ce-LEV-8 (F). The bars indicate the time period of the agonist application. Imax Pyr values (% of 100μM ACh response) are reported for each receptor above their respective representative recording traces. (TIF) [file ppat.1006996.s005.tif]

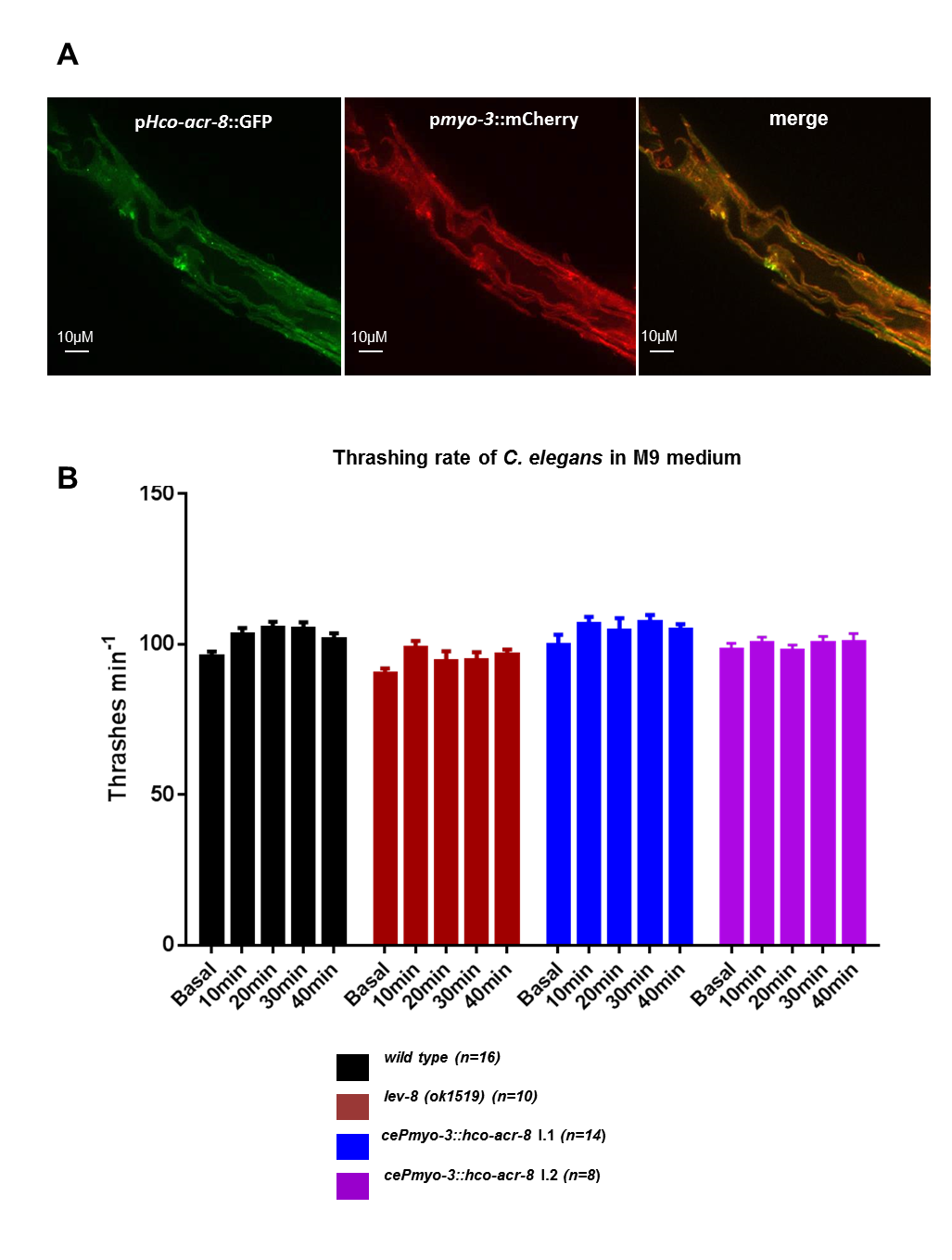

Supplement: S6 Fig — A. Expression patterns in C. elegans (N2) of GFP and mCherry driven by the H. contortus acr-8 and C. elegans myo-3 promoters respectively. B. Thrashing rate in M9 medium of C. elegans N2, lev-8(ok1519) and transgenic lev-8(ok1519) expressing Hco-ACR-8.A thrashing rate was established for wild type N2, lev-8(ok1519) and two lines of transgenic lev-8(ok1519); Pmyo-3::hco acr-8 C. elegans after 10, 20, 30 and 40 min in M9 medium. Data are the mean ± SEM. (TIF) [file ppat.1006996.s006.tif]

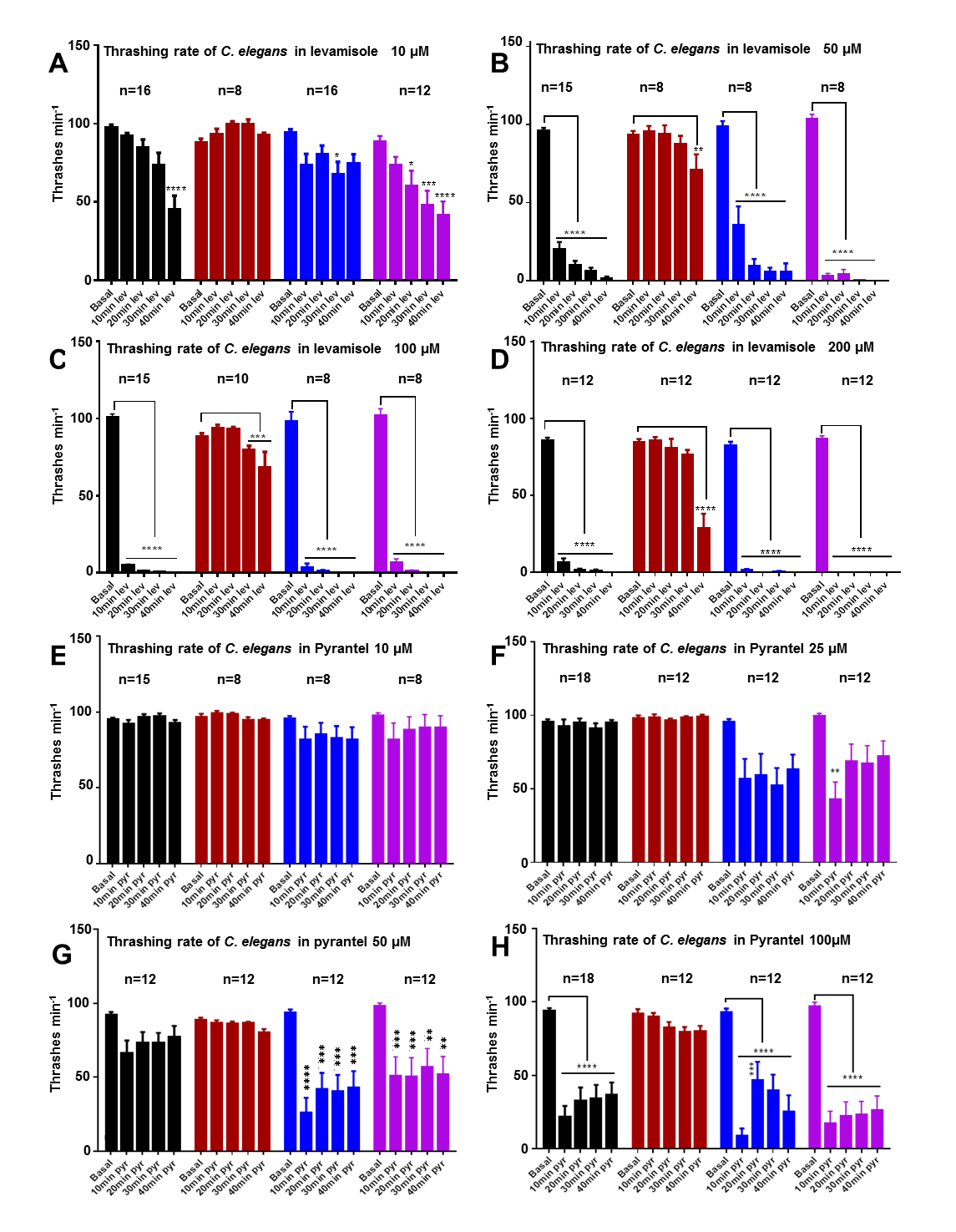

Supplement: S7 Fig — A thrashing rate was established for wild type N2, lev-8(ok1519) and two lines of transgenic lev-8(ok1519); Pmyo-3::hco acr-8 C. elegans after 10, 20, 30 and 40 min of exposure to Lev 10μM (A), 50μM (B), 100μM (C), 200μM (D) or Pyr 10μM (E), 25μM (F), 50μM (G) and 100μM (H) respectively. Basal thrashing rate was established after 10 min acclimatisation in M9 buffer. Data are the mean ± SEM of n ≥8, ****p<0.0001, ***p<0.001, **p<0.01 and *p<0.05, one way ANOVA with Bonferroni post-hoc test between basal and after drug treatment thrashing rate for the same strain. In black: wild-type N2 strain, in dark red: lev-8 (ok1519), in blue: cePmyo-3::hco-acr-8 l.1, in purple: cePmyo-3::hco-acr-8 l.2. (TIF) [file ppat.1006996.s007.tif]

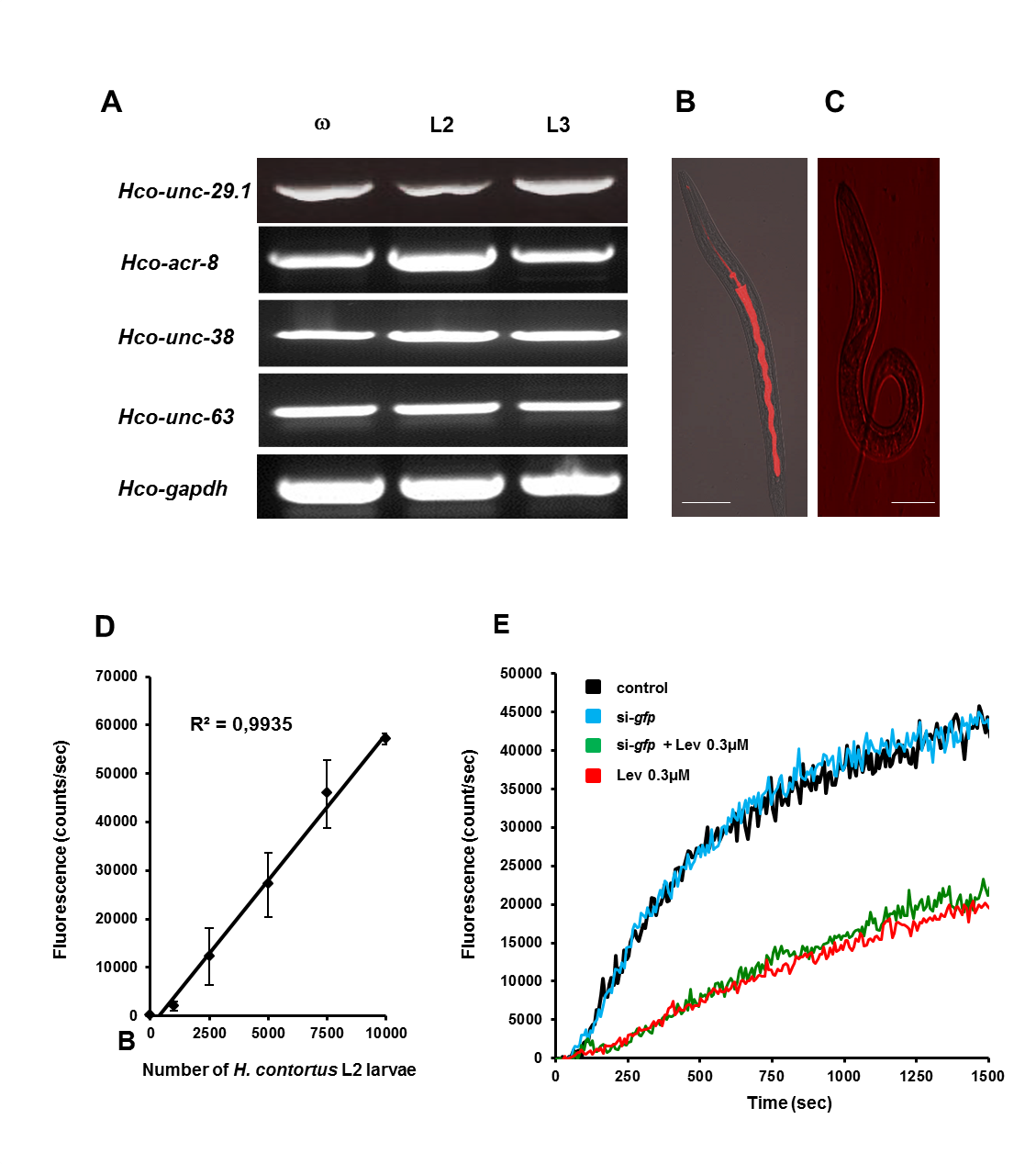

Supplement: S8 Fig — A. Expression of Hco-unc-29.1, Hco-acr-8, Hco-unc-63 and Hco-unc-38 in the free-living stages of Haemonchus contortus. Transcription of Hco-unc-29.1, Hco-acr-8, Hco-unc-63 and Hco-unc-38 throughout the free-living stages of H. contortus was investigated by RT-PCR; ω: embryonated egg; L2: second stage larvae; L3: third stage larvae. Integrity of cDNA preparations was verified by PCR using primers designed to amplify a fragment of the H. contortus gapdh cDNA. B-C. Monitoring of labelled siRNA ingestion by Haemonchus contortus second stage larvae. H. contortus L2 larvae incubated during 2 hours in a culture medium containing 1μM of non-specific siRNA (targeting gfp) labelled with Alexa 594 (B). Scale bar: 75μm. Negative control without fluorochrome added in the culture medium(C). Scale bar: 35μm. D. H. contortus L2 larvae migration assay using auto-fluorescence quantification. Correlation between the fluorescence counting (counts/sec) and the number of L2 larvae that migrated through the 30μM sieve during 25min. Migration assays were performed using 1000, 2500, 5000, 7500 and 10000 L2 larvae respectively. Each data point represents mean± SE of three independent runs. E. Non-target siRNA does not modulate motility / levamisole sensitivity of H. contortus L2 larvae. The automated larval migration assay (ALMA) was used to determine the putative impact of siRNA targeting gfp on migration or Lev sensitivity (0.3μM) of H. contortus L2 larvae. A. Representative recording traces of the real-time fluorescence counting relative to the L2 migration during 25min. Each trace corresponds to the mean data from 3 runs performed with 7500 L2 larvae. The control corresponds to untreated L2 larvae. (TIF) [file ppat.1006996.s008.tif]
