# Supplementary material for: Deciphering the molecular determinants of cholinergic anthelmintic sensitivity in nematodes: When novel functional validation approaches highlight major differences between the model Caenorhabditis elegans and parasitic species
Source: PLoS Pathog. 2018 May 2;14(5):e1006996. doi: 10.1371/journal.ppat.1006996 (PMC5931475; doi:10.1371/journal.ppat.1006996)
Supplement: S1 Table — Deduced amino-acid sequences from Cel-ACR-8 (Genbank accession number: JF416644.1) and Cel-LEV-8 (Genbank accession number: NM_077531.4) were used to perform a tBlastn search against a set of genomic databanks from nematodes available in WormBase Parasite (https://parasite.wormbase.org/). Nematode Clades as determined by Blaxter et al. [68].When available, full length cDNA sequences are indicated in bold: GB refers to Genbank accession number whereas WB refers to Worm base accession number. Life style abbreviations: VP: vertebrate parasite; IP: Insect parasite; FL: free-living. (DOCX) [file ppat.1006996.s009.docx]

|  | **Nematode species** | **Life- style** | **Database** | ***acr-8*** | **Reference** | ***lev-8*** | **reference** |
| --- | --- | --- | --- | --- | --- | --- | --- |
| **CLADE I** | ***Romanomermis culixivorax*** | IP | PRJEB1358 | NO |  | NO |  |
|  | ***Soboliphyme baturini*** | VP | PRJEB516 | NO |  | NO |  |
|  | ***Trichinella nativa*** | VP | PRJNA179527 | NO |  | NO |  |
|  | ***Trichinella spiralis*** | VP | PRJNA12603 | NO |  | NO |  |
|  | ***Trichuris muris*** | VP | PRJEB126 | NO |  | NO |  |
|  | ***Trichuris suis*** | VP | PRJNA179528 | NO |  | NO |  |
|  | ***Trichuris trichiura*** | VP | PRJEB535 | NO |  | NO |  |
| **CLADE III** | ***Acanthocheilonema viteae*** | VP | PRJEB4306 | YES | Scaff 00867 | NO |  |
|  | ***Anisakis simplex*** | VP | PRJEB496 | YES | Contig 0001341 | YES | Scaff 0004031/contig 0001137 |
|  | ***Ascaris lumbricoides*** | VP | PRJEB495 | YES | Scaff 0001028 | YES | Scaff 0000364 |
|  | ***Ascaris suum*** | VP | PRJNA 62057 | YES | **GB: KY654347** | YES | AG 00807 |
|  | ***Brugia malayi*** | VP | PRJNA10729 | YES | ChrX scaff 001 | NO |  |
|  | ***Brugia pahangi*** | VP | PRJEB497 | YES | Contig 0000295 | NO |  |
|  | ***Dirofilaria immitis*** | VP | PRJEB1797 | YES | **GB: KY654349** | NO |  |
|  | ***Dracunculus medinensis*** | VP | PRJEB500 | YES | Scaff 0000008 | NO |  |
|  | ***Elaeophora elaphi*** | VP | PRJEB502 | YES | Contig 0000109 | NO |  |
|  | ***Enterobius vermicularis*** | VP | PRJEB503 | YES | Scaff 0002849 | NO |  |
|  | ***Litomosoides sigmodontis*** | VP | PRJEB3075 | YES | Scaff 00254 | NO |  |
|  | ***Loa loa*** | VP | PRJNA24608 | YES | Scaff 7180000006887 | NO |  |
|  | ***Onchocerca flexuosa*** | VP | PRJEB512 | YES | 0001275 | NO |  |
|  | ***Onchocerca ochengi*** | VP | PRJEB1809 | YES | Scaff 00350 | NO |  |
|  | ***Onchocerca volvulus*** | VP | PRJEB513 | YES | Ovoc-OM1b | NO |  |
|  | ***Parascaris equorum*** | VP | PRJEB514 | YES | Scaff 0007048 | YES | Scaff 0012333 |
|  | ***Syphacia muris*** | VP | PRJEB524 | YES | Scaff 0000105 | NO |  |
|  | ***Toxocara canis*** | VP | PRJEB533 | YES | Scaff 0000002 | YES | Scaff 0000119 |
|  | ***Thelazia callipaeda*** | VP | PRJEB 1205 | YES | Scaff 0000013 | NO |  |
|  | ***Wuchereria bancrofti*** | VP | PRJEB536 | YES | Contig 0002189 | NO |  |
| **CLADE IV** | ***Bursaphelenchus xylophilus*** | PP | PRJEA64437 | YES | Scaff 00713 | NO |  |
|  | ***Globodera pallida*** | PP | PRJEB123 | YES | Scaff 654 | NO |  |
|  | ***Meloidogyne floridensis*** | pp | PRJEB6016 | YES | Scaff 00391 | NO |  |
|  | ***Meloidogyne hapla*** | PP | PRJNA29083 | YES | Contig 2 | NO |  |
|  | ***Meloidogyne incognita*** | PP | PRJEA28837 | YES | **GB : KY654350** | NO |  |
|  | ***Panagrellus redivivus*** | FL | PRJNA186477 | YES | KB455539 | NO |  |
|  | ***Parastrongyloides trichosuri*** | VP | PRJEB515 | YES | Scaff 0000001 | NO |  |
|  | ***Rhabditophanes sp.kr3021*** | FL | PRJEB1297 | YES | Scaff 0000025 | NO |  |
|  | ***Steinernema carpocapsae*** | IP | PRJNA202318 | YES | CARPO 882 | NO |  |
|  | ***Steinernema feltiae*** | IP | PRJNA204661 | YES | FELT 5692 | NO |  |
|  | ***Steinernema glaseri*** | IP | PRJNA204943 | YES | GLAS 6254 | NO |  |
|  | ***Steinernema monticolum*** | IP | PRJNA205067 | YES | MONTI 2762 | NO |  |
|  | ***Steinernema scapterisci*** | IP | PRJNA204942 | YES | SCAPT 971 | NO |  |
|  | ***Strongyloides papillosus*** | VP | PRJEB525 | YES | SPAL 0000082 | NO |  |
|  | ***Strongyloides ratti*** | VP | PRJEB125 | YES | SRAE Chr 1 | NO |  |
|  | ***Strongyloides stercoralis*** | VP | PRJEB528 | YES | SSTP scaff 0000015 | NO |  |
|  | ***Strongyloides venezuelensis*** | VP | PRJEB530 | YES | SVE 0000001 | NO |  |
| **CLADE V** | ***Ancylostoma caninum*** | VP | PRJNA72585 | YES | Contig 1048 | YES | Contig 294 |
|  | ***Ancylostoma ceylanicum*** | VP | PRJNA231479 | YES | Scaff 0072 | YES | Scaff 0103 |
|  | ***Ancylostoma duodenale*** | VP | PRJNA72581 | YES | Contig 219 | YES | Contig 219 |
|  | ***Angiostrongylus cantonensis*** | VP | PRJEB493 | YES | Contig 0001819 | NO |  |
|  | ***Angiostrongylus costaricensis*** | VP | PRJEB494 | YES | Contig0000505 | NO |  |
|  | ***Caenorhabditis angaria*** | FL | PRJNA51225 | YES | Cang 00065.g3190 | YES | Cang 00054 |
|  | ***Caenorhabditis brenneri*** | FL | PRJNA20035 | YES | **WB: CBN00977** | YES | **WB: CBN18834** |
|  | ***Caenorhabditis briggsae*** | FL | PRJNA10731 | YES | **WB: CBG17234** | YES | **WB: CBP04154** |
|  | ***Caenorhabditis japonica*** | FL | PRJNA12591 | YES | **WB: CJA08957** | YES | **WB: CJA01453** |
|  | ***Caenorhabditis remanei*** | FL | PRJNA53967 | YES | **WB : CRE07213** | YES | **WB: CRE07317** |
|  | ***Caenorhabditis sinica*** | FL | PRJNA194557 | YES | Scaff 00005 | YES | Scaff 00026 |
|  | ***Caenorhabditis tropicalis*** | FL | PRJNA53597 | YES | Scaff 0014533 | YES | Scaff 0021798 |
|  | ***Dictyocaulus_viviparus*** | VP | PRJEB5116 | YES | Contig 221 | NO |  |
|  | ***Heterorhabditis bacteriophora*** | IP | PRJNA13977 | YES | Scaffold 1054 | YES | Scaffold 1326 |
|  | ***Haemonchus contortus*** | VP | PRJEB506 | YES | **GB : ABV68891** | NO |  |
|  | ***Haemonchus placei*** | VP | PRJEB509 | YES | Scaff 0000250 | NO |  |
|  | ***Heligmosomoides bakeri*** | VP | PRJEB1203 | YES | scaff 0000822 | YES | Scaff 0001870 |
|  | ***Necator americanus*** | VP | PRJNA72135 | YES | KI657514 | YES | KI668926 |
|  | ***Nippostrongylus brasiliensis*** | VP | PRJEB511 | YES | Scaff 0000004 | NO |  |
|  | ***Oesophagostomum dentatum*** | VP | PRJNA72579 | YES | **GB : AFY08300** | YES | Contig 143 |
|  | ***Pristionchus expectatus*** | FL | PRJEB6009 | YES | Scaff 127 | YES | Scaff 381 |
|  | ***Pristionchus pacificus*** | FL | PRJNA12644 | YES | Contig 18 | YES | Contig 1 |
|  | ***Strongylus vulgaris*** | VP | PRJEB531 | YES | Scaff 0000704 | YES | Scaff 0024186 |
|  | ***Teladorsagia circumcincta*** | VP | PRJNA72569 | YES | **GB : HQ215517** | NO |  |

**S1 Table. Identification of Cel-*acr-8*and Cel *lev-8* homologs in nematode genomic data available in Wormbase-parasite databank**

Deduced amino-acid sequences from Cel-ACR-8 (Genbank accession number: JF416644.1) and Cel-LEV-8 (Genbank accession number: NM_077531.4) were used to perform a tBlastn search against a set of genomic databanks from nematodes available in WormBase Parasite ([http://parasite.wormbase.org/).](http://parasite.wormbase.org/).%20) Nematode Clades as determined by Blaxter *et al.* [68].When available, full length cDNA sequences are indicated in bold: GB refers to Genbank accession number whereas WB refers to Worm base accession number. Life style abbreviations: VP: vertebrate parasite; IP: Insect parasite; FL: free-living.
