# Supplementary material for: Deciphering the molecular determinants of cholinergic anthelmintic sensitivity in nematodes: When novel functional validation approaches highlight major differences between the model Caenorhabditis elegans and parasitic species
Source: PLoS Pathog. 2018 May 2;14(5):e1006996. doi: 10.1371/journal.ppat.1006996 (PMC5931475; doi:10.1371/journal.ppat.1006996)
Supplement: S2 Table — (DOCX) [file ppat.1006996.s010.docx]

| **PCR amplifications** | | | |
| --- | --- | --- | --- |
|  | **Primers** | **Forward (5'-3')** | **Reverse (5'-3')** |
| **RT-PCR** | Hco-unc-63 | TCTATTTGCCGTCGGACG | TCCTTCCTCTTATGTTCTGGTC |
|  | Hco-unc-38 | TGACATTATGTACTACCTGG | GGAGCTCACTCTCCTGAACG |
|  | Hco-acr-8 | GTGTGAACCACGAGACCTACA | TATCGTCCATGCTAGCTGGTT |
|  | Hco-unc-29 | ACTGCGCGAATGGAGCTGACT | TTGAGTGAGTAACTGACGCCA |
|  | Hco-gapdh | GTGTGAACCACGAGACCTACA | TATCGTCCATGCTAGCTGGTT |
| **cDNA**  **amplification** | Dim-acr-8 | ATGAGCGTATCACGATTACTG | TTATTGCACATATGATTGATT |
|  | Min-acr-8 | AGACATTATTAACATGCTTTT | TATCGAAATTCGACTGTGTCA |
|  | Asu-acr8 | ACCGGTGCGAGTCAACGAATG | TGGCAGCAGTGATGCCGCTCA |
|  | Hco-acr-8 | ATGCGTGCGTTCGGAATTGTT | CTATAAACCTTCAGAGTTCTT |
|  | Cel-acr-8 | ATGAACTTCGCATTTCTGCT | TCATTGATATTTGATGGGACA |
| **Promoter**  **amplification** | Hco-acr-8 | TTCACTGTCGCCATGGGTCAT | AAGCTTTCGGTGAAGTTAAAA |
| **Gene silencing experiments** | | | |
| **si-RNA duplex**  **(with dTdT 3’ overhang)** |  | **Sens (5'-3')** | **Antisens (5'-3‘)** |
|  | Hco-unc-63 | CGGACAAUGUUUCGUUCGU | ACGAACGAAACAUUGUCCG |
|  | Hco-unc-38 | CGUGCUCAGUAUGGUUAAA | UUUAACCAUACUGAGCACG |
|  | Hco-acr-8 | GGAUUCCGGAUAUCUUGCUUUAUAA | UUAUAAAGCAAGAUAUCCGGAAUCC |

**S2 Table. Sequences of primers and ds-siRNA**
